# Diabetic Retinopathy Screening Among Federally Qualified Health Center Patients Using Point-of-Care AI: DRES-POCAI: A Trial Protocol

**DOI:** 10.1001/jamanetworkopen.2025.38114

**Published:** 2025-10-21

**Authors:** Edgar A. Diaz, Marva L. Seifert, Vida Gruning, Nicole A. Stadnick, Elizabeth Lugo-Butler, Ariel N. Servin, Christian I. Rodríguez-Rosales, Carrie Geremia, Chaithanya Ramachandra, Malavika Bhaskaranand, Dan Howard, Oliver Solis, Sharon Velasquez, Brian Snook, Sonia Tucker, Fatima A. Muñoz

**Affiliations:** 1 San Ysidro Health, San Diego, California; 2 Department of Medicine, University of California San Diego, La Jolla; 3 Department of Psychiatry, University of California San Diego, La Jolla; 4 University of California San Diego Altman Clinical and Translational Research Institute Dissemination and Implementation Science Center, La Jolla; 5 Child and Adolescent Services Research Center, San Diego, California; 6 Eyenuk Inc, Woodland Hills, California

## Abstract

**Importance:**

Diabetic retinopathy screening (DRS) rates have historically been low among underserved populations due to barriers in accessing traditional eye care. Although artificial intelligence (AI)–powered DRS provides a potential strategy to improve screening rates, its optimal integration into primary care workflows within federally qualified health centers (FQHCs) requires rigorous evaluation. The clinical workflow of the Diabetic Retinopathy Screening Point-of-Care Artificial Intelligence (DRES-POCAI) trial in FQHCs integrates AI-powered DRS with electronic health records (EHRs) to automate results and prompt referrals, aiming to improve screening rates and facilitate early diagnosis and timely treatment.

**Objective:**

To increase DRS rates, facilitate early-stage DR detection, improve timely eye specialist follow-up, and assess the effect of DRS on patients’ knowledge, attitudes, self-efficacy, and satisfaction.

**Design, Setting, and Participants:**

DRES-POCAI is a patient-level, multiclinic, open-label, parallel superiority randomized clinical trial at 2 FQHC sites of San Ysidro Health in San Diego County, California. The study recruitment targets 848 active FQHC patients aged 22 years or older with diabetes, no DRS in the prior 11 months, and scheduled medical visits during the intervention period. Patients with a history of retinopathy or retinal vascular occlusion and other physical or mental conditions are excluded. The study started in June 2024, with recruitment anticipated to conclude in August 2025 and follow-up until February 2026.

**Intervention:**

The intervention arm receives DRS at their primary care clinic using an AI-powered DRS system, with retinal image analysis to identify more than mild DR and vision-threatening DR. Results are immediately available in the EHRs, and practitioners receive risk-stratified referral recommendations. The usual care arm receives referrals to an FQHC optometrist or external eye care practitioner, with results transmitted to the medical home later.

**Main Outcomes and Measures:**

The primary outcome is DRS completion status. Secondary outcomes include DR diagnosis stage, specialist referrals, and participants’ DR knowledge, attitudes, and intentions regarding future AI-powered DRS.

**Results:**

Findings will be disseminated in peer-reviewed publications after data collection and analysis.

**Conclusions and Relevance:**

DRES-POCAI will determine the effectiveness of an AI-powered DRS intervention to increase DRS rates in FQHC primary care workflows.

**Trial Registration:**

ClinicalTrials.gov Identifier: NCT06721351.

## Key Points

**Question:**

How can artificial intelligence (AI)–powered point-of-care diabetic retinopathy screenings in federally qualified health centers improve access for medically underserved patients?

**Findings:**

The Diabetic Retinopathy Screening Point-of-Care Artificial Intelligence trial aims to demonstrate that a multicomponent approach—AI-powered diabetic retinopathy screenings, real-time integration of results with electronic health records, and patient education—within federally qualified health centers can improve patient adherence to annual retinal screening and diabetes standard of care.

**Meaning:**

The Diabetic Retinopathy Screening Point-of-Care Artificial Intelligence trial aims to establish a replicable protocol and transform clinical workflows to enhance access to diabetes eye care by accelerating AI integration in clinical settings, ultimately improving patient care, population health, health care costs, and patient and practitioner experience.

## Teaser

This trial protocol describes a multicomponent randomized clinical trial that integrated an artificial intelligence (AI)–powered diabetic retinopathy screening workflow to improve screening rates, detection of early-stage disease, and timely eye specialist follow-up.

## Introduction

Standards of care for individuals with diabetes include annual diabetic retinopathy screening (DRS)^1^; however, DRS is frequently performed outside primary care clinics, requiring referrals to eye care practitioners.^2,3^ This fragmented approach is particularly evident in federally qualified health centers (FQHCs), where less than one-third offer in-clinic eye care services.^4^ Patients served by FQHCs frequently face barriers to care, including lack of insurance, financial constraints, transportation challenges, and limited health literacy, which contribute to low DRS rates.^5,6^ The current referral process for DRS is inefficient, creating additional burdens, such as increased wait times, difficulties in navigating referrals, insurance complexities, appointment scheduling challenges, and fragmentation of patient care.^7,8^ These challenges limit the primary care practitioner’s (PCP’s) ability to make informed clinical decisions while coordinating advanced, comprehensive care with specialists.^8,9^

DR is the leading cause of blindness among the working-age population.^10,11^ In the US, approximately 38.4 million people have diabetes; of those, an estimated 26.4% have DR^11,12^ and 5.1% develop vision-threatening DR (vtDR).^11^ Black and Hispanic individuals have a higher standardized prevalence of vtDR (8.7% and 7.1%, respectively) than White individuals (3.6%).^11,13^ The prevalence of DR and its progression to severe advanced stages increases with poor diabetes management, comorbidities, and age.^11,14,15,16^ DRS rates vary widely in the literature, ranging from 11% to 71%,^17,18^ with lower rates observed in minoritized race and ethnicity populations.^19^ This low screening rate increases the risk of delayed diagnosis because DR often presents with no symptoms in its early stages, when treatment is most effective.^20,21^

Artificial intelligence (AI)–powered DRS (AI-DRS) systems have been implemented in various global health care settings.^22,23,24^ In the US, AI-DRS implementation lacks a unified approach, with applications variably adopted across settings, such as laboratory patient service centers, endocrinology and primary care practices, and FQHCs.^25,26,27^ Serving 32.5 million patients nationwide,^28^ FQHCs can provide valuable insights into the potential of AI technology to address care gaps, enhance point-of-care (POC) preventive screenings, and improve patient outcomes.

Although AI-DRS have shown promise in improving screening rates and DR detection,^17^ gaps remain in knowledge and literature regarding the optimal use and integration of diagnostic AI into primary care clinical workflows,^29,30^ given the evolving nature of health care AI and its rapid pace of development.^29,31,32^ The Diabetic Retinopathy Screening Point-of-Care Artificial Intelligence (DRES-POCAI) trial aims to address this gap by detailing the integration of an AI-DRS system for POC DRS into FQHC clinical workflows, using a multicomponent approach to reduce access barriers for medically underserved populations. This integration facilitates access to DRS in the patient’s medical home, improves the PCP’s clinical decision-making process, and provides immediate transmission of the results into the patient’s electronic health records (EHRs) for prompted referrals to the eye specialist based on DRS results.

## Methods

### Study Design

**Figure 1.**
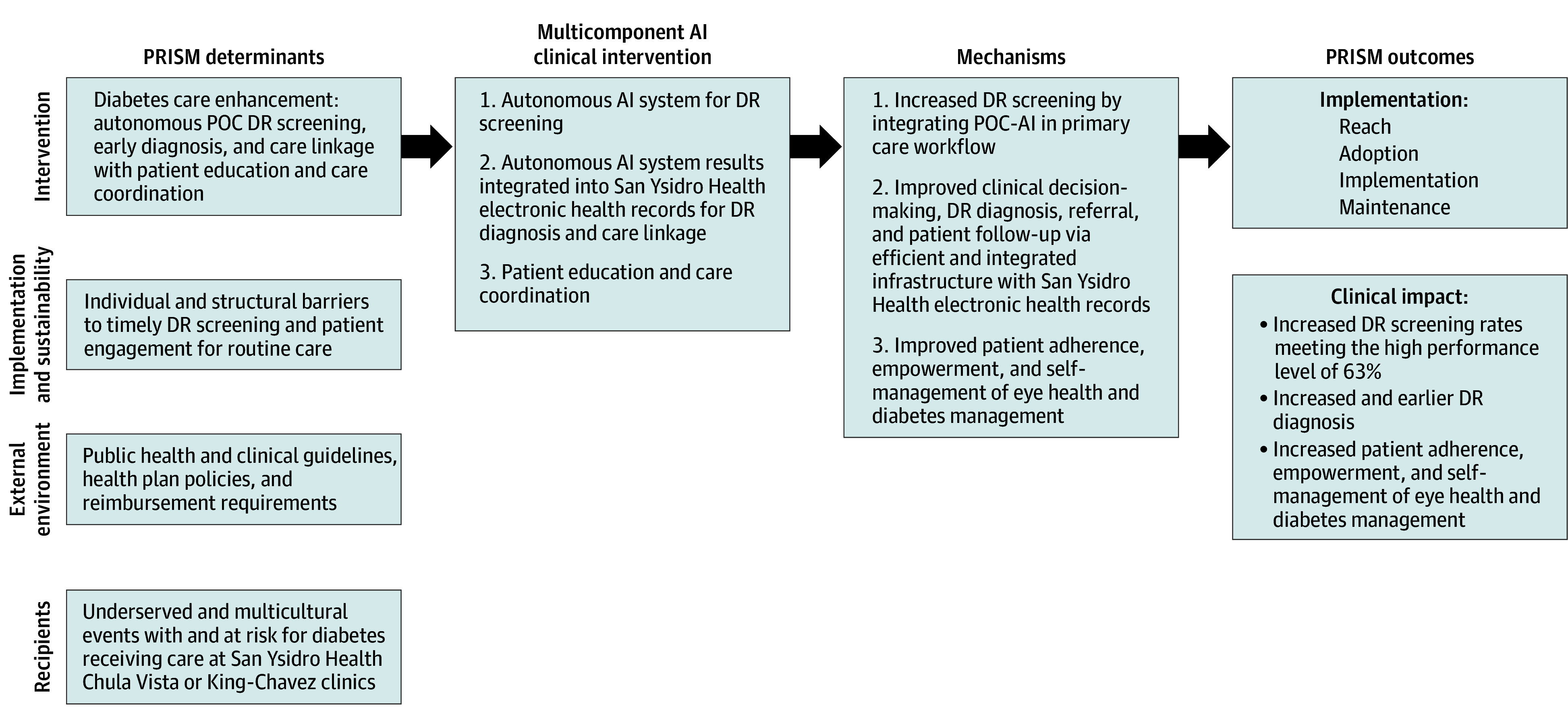
Pragmatic Robust Implementation and Sustainability Model (PRISM) Framework for the Diabetic Retinopathy Screening Point-of-Care Artificial Intelligence (DRES-POCAI) Trial The PRISM framework as applied to the DRES-POCAI study, outlining the multicomponent artificial intelligence (AI)–powered point-of-care (POC) diabetic retinopathy (DR) screening intervention, relevant PRISM determinants (recipients, external environment, implementation, and sustainability infrastructure), and the mechanisms driving implementation and clinical outcomes.

The DRES-POCAI study is a multicomponent clinical intervention using a controlled, open-label, parallel superiority randomized clinical trial design involving patient-level randomization to the intervention (AI-DRS) arm or the usual care (UC; referral to an eye specialist for DRS) arm to evaluate the impact of an AI screening tool on DRS uptake and diagnosis. The study uses the Pragmatic Robust Implementation and Sustainability Model (PRISM) to refine, test, and evaluate the multicomponent clinical intervention.^33,34^ PRISM offers a multilevel conceptualization of context: recipient (patient and practitioner characteristics), intervention characteristics, implementation and sustainability infrastructure within FQHCs, clinical referrals, external environment (public health and clinical guidelines), health plans, and reimbursement considerations (Figure 1). This trial protocol follows the Standard Protocol Items: Recommendations for Interventional Trials (SPIRIT) reporting guideline.

### Algorithm Description

DRES-POCAI uses EyeArt (Eyenuk Inc), the first FDA-cleared AI-DRS system, to detect both more than mild DR (mtmDR) and vtDR. This AI-DRS system was developed using 375 000 images, tested on more than 850 000 images,^35^ and validated in a prospective, multicenter, pivotal clinical trial of 942 individuals with diabetes,^36,37^ demonstrating high accuracy against the Early Treatment Diabetic Retinopathy Study reference standard. Specifically, the sensitivity for mtmDR was 96% (specificity, 88%), and the sensitivity for vtDR was 97% (specificity, 90%).^36^ The AI-DRS system provided conclusive reports for more than 97% of eyes, with most images obtained without dilation. In a retrospective study of more than 100 000 consecutive encounters with people with diabetes, 91.3% sensitivity and 91.1% specificity were achieved in detecting mtmDR.^35,38^ The AI-DRS system’s algorithms will remain static throughout the DRES-POCAI evaluation process and not be retrained. DRES-POCAI uses a patient-centered model with trained clinical staff to guide appropriate patient care and follow-up based on AI- DRS results for all 4 outcomes: (1) mtmDR positive, vtDR positive; (2) mtmDR positive, vtDR negative; (3) mtmDR negative, vtDR negative; and (4) ungradable.

### Integration of Autonomous AI-DRS Into the Primary Care Workflow

**Figure 2.**
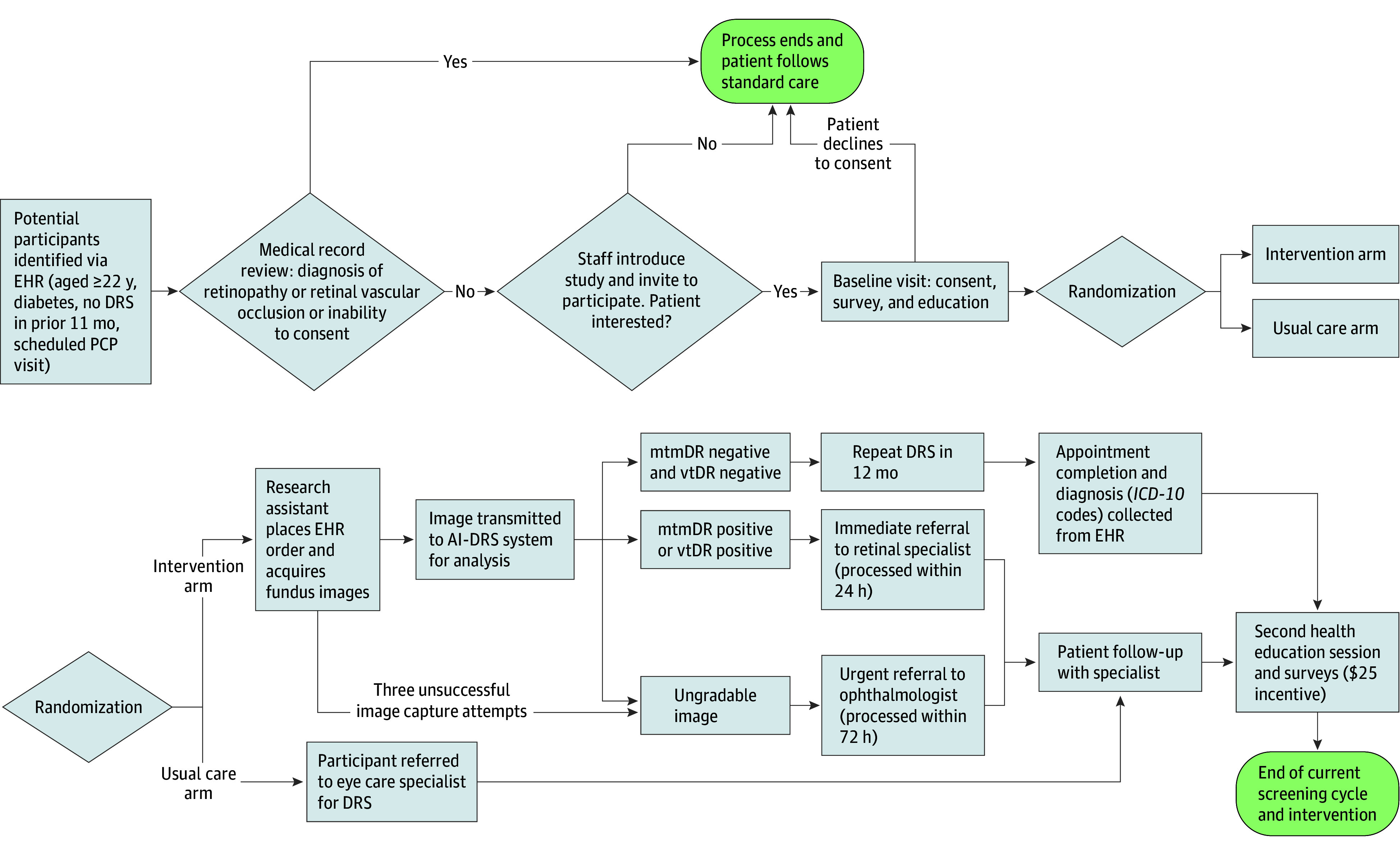
Diabetic Retinopathy Screening (DRS) Point-of-Care Artificial Intelligence (AI) Participant Workflow Participants identified via electronic health record (EHR) undergo eligibility screening. Consenting participants are randomized to the intervention arm (AI-powered DRS [AI-DRS]) or the usual care arm (standard referral for DRS). The intervention arm involves AI-DRS image acquisition and analysis, leading to risk-stratified referrals (immediate referral to a retina specialist for more than mild DR [mtmDR] positive or vision-threatening DR [vtDR] positive; urgent referral to an ophthalmologist for ungradable images) or repeat screening in 12 months (mtmDR negative and vtDR negative). Both study arms include appointment completion tracking and a second health education session. *ICD-10* indicates *International Statistical Classification of Diseases and Related Health Problems, Tenth Revision*; PCP, primary care practitioner.

DRES-POCAI integrates autonomous DRS into the primary care workflow using the AI-DRS system and the a nonmydriatic retinal camera (Topcon TRC-NW400, Topcon Healthcare), operated by trained research assistants in an FQHC setting. A 2-day operator training program covers system setup, basic clinical review, the AI-DRS system’s software, hands-on screening practice, competency checks, and troubleshooting. The workflow (Figure 2) initiates with the research assistant generating a screening order within the Epic EHR (Epic Systems), which is sent to the AI-DRS system’s server. The operator then uses the AI-DRS system’s desktop client to select the order, acquire fundus images, and transmit them to the server for analysis. The AI-DRS system provides real-time image quality feedback during image acquisition; 3 unsuccessful attempts yield an ungradable result.

The AI-DRS system’s server analyzes the images and provides DRS results. These results are then transmitted to the EHR system, triggering risk-based stratified referrals and prompting PCPs for review and approval. Patients with positive results for vtDR or mtmDR are given an immediate referral (processed within 24 hours) to a retina specialist. Patients with ungradable images, which may often indicate underlying pathology, are given an urgent referral (processed within 72 hours) to an ophthalmologist. Patients with negative results for both mtmDR and vtDR are scheduled for repeat DRS in 12 months.

### Integration of AI-DRS and EHR Systems

**Figure 3.**
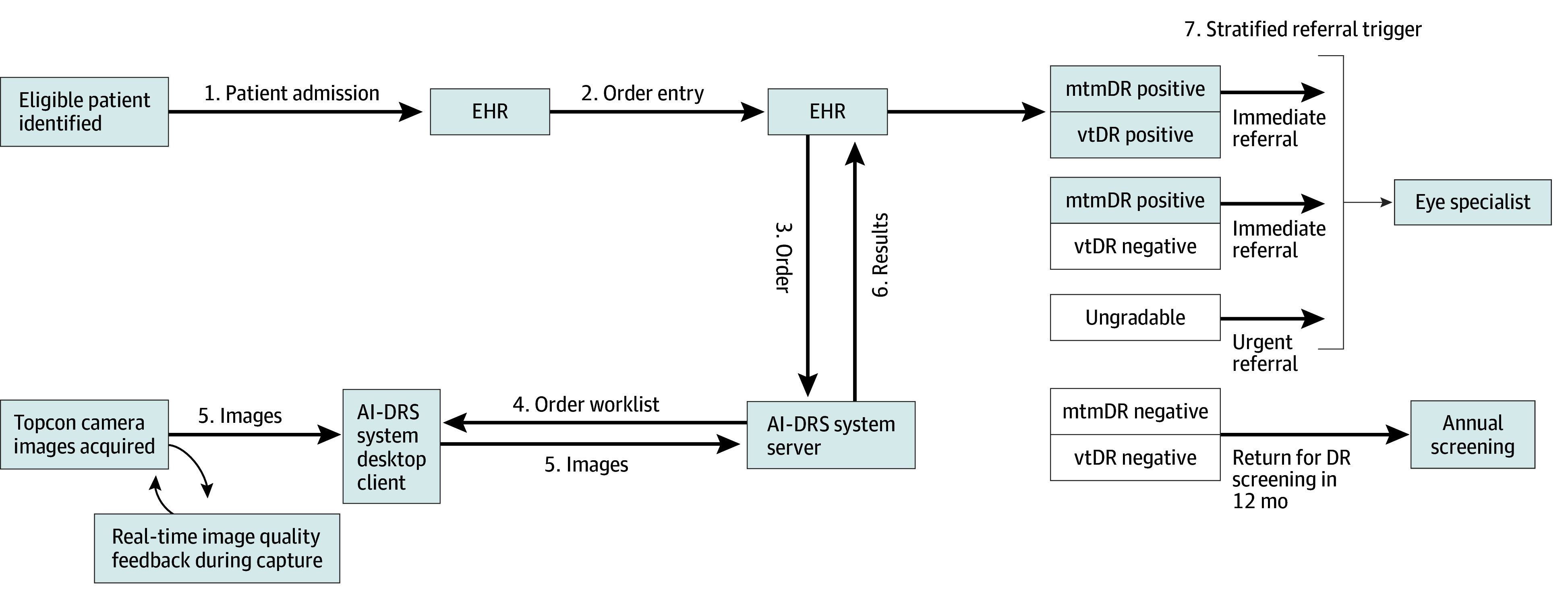
Diabetic Retinopathy Screening (DRS) Point-of-Care Artificial Intelligence (AI) System and Electronic Health Record (EHR) Integration Workflow Process initiation via patient admission and order entry in the EHRs. The EHR transmits a health level 7 order message to the AI-DRS system server. Retinal images, acquired by the AI-DRS desktop client and camera with real-time image quality feedback, are sent to the server for analysis. Artificial intelligence–generated results are returned as a health level 7 observation result message to the EHR, triggering stratified referrals: immediate referrals for more than mild diabetic retinopathy (mtmDR) positive or vision-threatening diabetic retinopathy (vtDR) positive results, urgent referrals for ungradable images, or repeat diabetic retinopathy screening in 12 months for negative results.

DRES-POCAI integrates the AI-DRS system with the FQHC’s EHR to improve patient care by leveraging system interoperability, enhancing data access, reducing health care costs, and ensuring robust data security (Figure 3). The bidirectional integration uses Health Level 7^39,40^ standard message types for efficient communication between the FQHC’s EHR and the AI-DRS system’s server and additional support for other formats (eg, JSON and PDF) as needed. The EHR sends outbound order messages to the server, whereas inbound observation result messages provide diagnostic reporting back to the EHR. These transactions are brokered via the LKTransfer Interface Engine (ELLKAY LLC), enabling seamless review of AI-generated screening reports, assignment of referral pathways, and integration of imaging studies into the PACS (Picture Archiving and Communication System) systems.^41^ This interoperable framework ensures the streamlined incorporation of AI-derived diagnostic data into clinical workflows while maintaining practitioner oversight and facilitating downstream ophthalmic care coordination.

### Study Population

The study population consists of active patients from 2 participating clinics of San Ysidro Health, an FQHC in San Diego County, California. Eligibility criteria included patients with diabetes who receive medical care in one of DRES-POCAI’s research clinic sites, are 22 years or older, have not had a DRS within the preceding 11 months, have a medical visit scheduled during the intervention period, and can read and understand English or Spanish to provide informed consent and complete study surveys. Exclusion criteria align with the authorized use of the AI-DRS system: prior diagnosis of DR, macular edema, or retinal vascular occlusion; persistent visual impairment in one or both eyes; history of ocular injections, retinal laser treatment, or intraocular surgery (excluding cataract surgery); pregnancy; or diagnosis of mental or degenerative disease that precludes self-consent.

### Codesign Phase and Protocol Refinement

Before implementation, the DRES-POCAI team conducted a codesign phase, engaging FQHC patients, clinicians, and staff to identify potential barriers to program success and refine the intervention’s clinical and implementation protocol. The codesign identified potential failures and solutions that were evaluated and integrated into the study protocol and clinical implementation.

### Randomization, Allocation, and Intervention Description

DRES-POCAI uses individual-level randomization for group assignments. Study staff generate a recruitment report from the EHR, listing patients from the 2 FQHC clinics who meet the eligibility criteria. Study staff contact potential participants to introduce the study and schedule an on-site baseline visit. During this visit, trained bilingual staff explain the study in detail and facilitate the informed consent, surveys, and the first education session in the participants’ preferred language (English or Spanish). After completing these steps, participants are randomized and assigned to the intervention or UC arm using a randomization app.^42^

For the intervention group, DRS is conducted during the baseline visit using the AI-DRS system. Results are automatically uploaded into the EHR, and a copy is provided to the participant. The EHR automatically generates risk-based stratified referrals to an eye specialist (ophthalmologist or retina specialist) for positive or ungradable results and prompts PCPs for review and approval. For the UC arm, the participant receives a referral to the eye care specialist for a DRS according to the standard clinical guidelines. Participant navigation support for appointment booking is provided to both groups.

### Effect and Outcome Measures

DRES-POCAI evaluates DRS rates, DR diagnosis, and patient education outcomes. Participant-level data, including demographics, clinical data, and prior screening history, are collected from the FQHC’s EHR at enrollment, 90 days after the baseline visit (primary outcome), and 180 days after the baseline visit (secondary outcome). Secondary outcomes are (1) AI-DRS system results (reported as normal, mtmDR, vtDR, or ungradable), (2) referrals to an eye specialist, and (3) DR diagnosis, using *International Statistical Classification of Diseases and Related Health Problems, Tenth Revision (ICD-10)* codes abstracted from the EHR and manually validated by study staff (Table).

**Table.** Screening Results From the Artificial Intelligence–Powered Diabetic Retinopathy Screening System Mapped to Their Corresponding Formal Diagnostic Codes^a^

*ICD-10* code by examination result	Diseases
**Nonproliferative diabetic retinopathy **	
mtmDR negative, vtDR negative	
E10.9	Type 1 diabetes without complications
E11.9	Type 2 diabetes without complications
E10.329	Type 1 diabetes with mild nonproliferative diabetic retinopathy without macular edema
E11.329	Type 2 diabetes with mild nonproliferative diabetic retinopathy without macular edema
E08.329	Diabetes due to underlying condition with mild nonproliferative diabetic retinopathy without macular edema
mtmDR positive, vtDR negative	
E10.339	Type 1 diabetes with moderate nonproliferative diabetic retinopathy without macular edema
E11.339	Type 2 diabetes with moderate nonproliferative diabetic retinopathy without macular edema
E08.339	Diabetes due to underlying condition with moderate nonproliferative diabetic retinopathy without macular edema
mtmDR positive, vtDR positive	
E10.321	Type 1 diabetes with mild nonproliferative diabetic retinopathy with macular edema
E11.321	Type 2 diabetes with mild nonproliferative diabetic retinopathy with macular edema
E08.321	Diabetes due to underlying condition with mild nonproliferative diabetic retinopathy with macular edema
E10.331	Type 1 diabetes with moderate nonproliferative diabetic retinopathy with macular edema
E11.331	Type 2 diabetes with moderate nonproliferative diabetic retinopathy with macular edema
E08.331	Diabetes due to underlying condition with moderate nonproliferative diabetic retinopathy with macular edema
E10.341	Type 1 diabetes with severe nonproliferative diabetic retinopathy with macular edema
E11.341	Type 2 diabetes with severe nonproliferative diabetic retinopathy with macular edema
E08.341	Diabetes due to underlying condition with severe nonproliferative diabetic retinopathy with macular edema
E10.349	Type 1 diabetes with severe nonproliferative diabetic retinopathy without macular edema
E11.349	Type 2 diabetes with severe nonproliferative diabetic retinopathy without macular edema
E08.349	Diabetes due to underlying condition with severe nonproliferative diabetic retinopathy without macular edema
**Proliferative diabetic retinopathy **	
mtmDR positive, vtDR positive	
E10.351	Type 1 diabetes with proliferative diabetic retinopathy with macular edema
E11.351	Type 2 diabetes with proliferative diabetic retinopathy with macular edema
E08.351	Diabetes due to underlying condition with proliferative diabetic retinopathy with macular edema
E10.359	Type 1 diabetes with proliferative diabetic retinopathy without macular edema
E11.359	Type 2 diabetes with proliferative diabetic retinopathy without macular edema
E08.359	Diabetes due to underlying condition with proliferative diabetic retinopathy without macular edema

Abbreviations: *ICD-10*, *International Statistical Classification of Diseases and Related Health Problems, Tenth Revision*; mtmDR, more than mild diabetic retinopathy; vtDR, vision-threatening diabetic retinopathy.

^a^ A direct comparison of paired results is only possible for participants in the intervention arm who receive abnormal or ungradable screening results and complete a follow-up examination with an eye specialist.

### Additional Measures

The effect and efficiency of the AI-DRS system are evaluated by collecting data on the number of screening orders submitted, screenings completed, ungradable results and attempts, and the time participants spend in front of the camera. Participants’ knowledge, attitudes, self-efficacy, and satisfaction are assessed at baseline and 6-month follow-up using a 15-minute questionnaire^43^ refined and tested during the study’s codesign phase. The questionnaire evaluates participants’ knowledge and attitudes about DR (eg, “Do you think your diabetes can make you blind?”), self-efficacy (eg, “I am confident that I can take care of my eyes”), comfort and trust in the POC AI-DRS system, and overall satisfaction with the intervention. Additional data, including sex, date of birth, socioeconomic status (eg, educational level and insurance status), marital status, ethnicity, access and barriers to health care (social determinants of health), personal and family history of diabetes, smoking and alcohol intake, and clinical data (eg, anthropometry, blood pressure, hemoglobin A_1c_ level, lipid levels, and annual kidney health evaluation [estimated glomerular filtration rate or urine albumin-creatinine ratio]), are obtained from study questionnaires at baseline and EHR abstraction and used to describe the study population.

### Statistical Analysis

#### General Approach

A detailed trial protocol and statistical analysis plan are provided in Supplement 1. Participant characteristics will be described using descriptive statistics. Categorical data will be presented as numbers (percentages), and continuous data will be presented as means (SDs) or medians (IQRs), depending on distribution. No hypothesis testing will be used to compare the baseline characteristics between the intervention and UC arms.

#### Primary and Secondary Outcomes

The effect of the intervention will be assessed by comparing the primary and secondary outcomes between the intervention and UC arms using logistic or Poisson regression (depending on the distribution of the outcome), adjusting for identified covariates, including study clinic site, with the study intervention arm as the primary independent variable. A 2-sided significance level of *P* < .05 will be used, and 95% CIs will be reported. Covariate selection will aim for a parsimonious model to achieve clarity and avoid overfitting and to ensure inclusion of biologically and statistically relevant variables. The Hosmer and Lemeshow covariate inclusion approach (purposeful selection at each modeling step) will be used to develop models.^44^ Data will be checked for normality of distribution prior to analysis. If assumptions are violated, appropriate transformations will be applied. Clinic site, sex, age, race and ethnicity, and clinical risk factors will be assessed as covariates in both the primary (DRS completion) and secondary (DR diagnosis) initial regression models. Additional system data will be collected to describe intervention implementation and workflow, including the number of orders submitted, screenings completed, number of ungradable results and attempts, and time spent by the patient in front of the camera.

Participant knowledge, attitudes, self-efficacy, and satisfaction will be assessed using a questionnaire. Changes in these end points will be evaluated using a difference-in-differences analysis to evaluate the impact of the intervention on participant knowledge, attitudes, self-efficacy, and satisfaction using a pre/post design.

#### Safety End Points

No formal safety end points will be analyzed. However, all documented adverse events will be recorded and presented in a table in the final report, detailing the date, description, resolution, follow-up, and outcome. To ensure participant safety and study integrity, the Data Safety Monitoring Committee will meet quarterly to review recruitment progress, data quality, and protocol adherence and to advise on managing any special circumstances.

#### Sample Size and Power Considerations

Preliminary analysis among patients with active diabetes from the 2 clinics established that approximately 59% had completed DRS during the previous year. We hypothesize that intervention arm participants will increase DRS completion by 10 percentage points, from 59% to 69%. Assuming the UC arm participants will maintain a similar retinal screening completion rate (59%) as during the initial assessment, with α = .05 (significance) and β = 0.8 (power), the target enrollment for analysis is 722 participants (361 per arm). Anticipating an approximate 15% attrition rate, the target enrollment will be 848 patients (424 per arm) to ensure an analysis size of 722 participants (361 per arm) for a 2-tailed, independent-sample Pearson χ^2^ test. No interim analyses were planned for this study.

#### Data Management

DRES-POCAI uses EHR reports to identify eligible patients. Reports include demographics, site, PCP, appointment, diabetes diagnosis, and quality indicators (eg, hemoglobin A_1c_ level and DRS). To determine study eligibility, patient medical record reviews of scanned optometry and ophthalmology reports that indicate DR diagnosis or DRS are conducted in EHRs. REDCap (Research Electronic Data Capture) is the study’s data management system that documents eligibility assessments, enrollment outcomes, consent, survey, and participant navigation. DRS completion and eyecare diagnoses (*ICD-10* codes) are extracted from the EHR as part of the enrolled participant report.

Data use agreements govern data sharing with research partners. UC San Diego (evaluation partner) receives only deidentified data, and Eyenuk (AI technology partner) receives only aggregated data. Aggregate enrollment, outcomes, and protocol deviations reports are sent quarterly to the Data Safety Monitoring Committee for review and recommendations. UC San Diego and the FQHC’s institutional review boards reviewed and approved the study.

### Dissemination of Trial Findings

Study findings will be disseminated through publication in a peer-reviewed journal and submission to ClinicalTrials.gov. The study protocol and statistical code will be made publicly available. Authorship will adhere to International Committee of Medical Journal Editors standards. Additionally, results will be shared with participants and clinicians via a newsletter, with FQHC leadership, and through a policy brief for dissemination to payers and other FQHCs.

### Data Sharing Plan

The deidentified dataset will be available to external investigators on approval of a scientific request. These requests must adhere to established FQHC guidelines and will be presented to the FQHC’s research review committee, which will assess the proposed research for scientific merit and ethical considerations.

## Discussion

The DRES-POCAI study seeks to address barriers to DRS and early diagnosis among underserved populations with diabetes in FQHC settings. These populations often face limited resources, limited academic attainment, transportation challenges, and difficulties navigating referrals and follow-up appointments, hindering their access to necessary screenings and care. These barriers align with these groups’ historically low DRS and follow-up rates.^45^

DRES-POCAI aligns with the Quadruple Aim of health care^46^: enhancing patient experience, improving population health, reducing health care costs, and promoting care team well-being.^45^ Specifically, by providing a more convenient alternative to traditional DRS (reducing lost work or wages and long delays in obtaining results) and streamlining referrals,^47,48,49^ DRES-POCAI has the potential to improve efficiency, reduce health care costs for FQHCs, and lessen burnout among FQHC care teams.

This study will evaluate the effect of a bundle intervention integrating a POC AI-DRS system into the primary care workflow at FQHCs. This intervention offers a substantial advantage for this population by providing a readily accessible screening tool within the primary care setting, thereby reducing the burden of additional appointments and mitigating barriers to care access. The immediate availability of results and automated, severity-stratified referrals to specialists will expedite the identification of at-risk patients and facilitate their timely linkage to appropriate care,^50,51^ potentially reducing complications and improving quality of life. By expediting diagnosis and referrals, the POC process can potentially improve quality metrics and supports a value-based care model.^52^

### Limitations

This study has several limitations. DRES-POCAI’s reliance on Epic to integrate screening results and referrals and the costs associated with equipment, licensing, and trained personnel may limit generalizability, particularly for smaller FQHCs. The generalizability of the results may also be limited by the characteristics of the populations served by specific FQHCs, which can vary substantially, particularly in rural areas where access to higher levels of care may be challenging. Also, the implementation of DRES-POCAI occurs during a period when AI adoption in health care is still relatively new, potentially necessitating a multicomponent intervention (eg, patient education) to encourage the uptake of AI-DRS. Differences in the age distribution and specific needs and barriers of the population served by specific FQHCs can affect the program’s implementation and increase the proportion of ungradable patients due to underlying pathologies, which can reduce the effectiveness of the screening. Additionally, incorporating research-specific activities, such as formal eligibility and consent processes, alongside supportive elements, such as patient navigation, may introduce selection bias and create conditions that differ from a standard clinical workflow, potentially limiting the direct applicability of these findings to routine practice where such components would not exist. A subsequent quality improvement phase focused on pure clinical implementation without these research components could provide additional insights into community-based implementations.

## Conclusions

DRES-POCAI aims to determine whether an AI-DRS system can be safely and effectively integrated into primary care settings with the proper clinical workflow and educational component. DRES-POCAI is unique in evaluating this integration into FQHC settings and using automated, severity-stratified referrals. This approach can potentially improve clinical workflows and patient outcomes and inform future strategies for deploying AI in similar health care environments.

## Notes

Supplement 1.
Trial Protocol and Statistical Analysis Plan

Supplement 2.
Data Sharing Statement

## References

[1] American Diabetes Association Professional Practice Committee. 12. Retinopathy, neuropathy, and foot care: standards of care in diabetes-2024. Diabetes Care. 2024;47(suppl 1):S231-S243. doi:10.2337/dc24-S012 38078577 PMC10725803

[2] Kummerle D, Beals D, Simon L, Rogers F, Pogroszewski S. Revolutionizing diabetic retinopathy screening: integrating AI-based retinal imaging in primary care. J CME. 2025;14(1):2437294. doi:10.1080/28338073.2024.2437294 39776444 PMC11703125

[3] Liu J, Gibson E, Ramchal S, . Diabetic retinopathy screening with automated retinal image analysis in a primary care setting improves adherence to ophthalmic care. Ophthalmol Retina. 2021;5(1):71-77. doi:10.1016/j.oret.2020.06.016 32562885 PMC8546907

[4] Woodward MA, Hicks PM, Harris-Nwanyanwu K, ; American Academy of Ophthalmology Taskforce on Ophthalmology and Community Health Centers. Eye care in federally qualified health centers. Ophthalmology. 2024;131(10):1225-1233. doi:10.1016/j.ophtha.2024.04.019 38697267 PMC11416322

[5] Bastos de Carvalho A, Lee Ware S, Belcher T, . Evaluation of multi-level barriers and facilitators in a large diabetic retinopathy screening program in federally qualified health centers: a qualitative study. Implement Sci Commun. 2021;2(1):54. doi:10.1186/s43058-021-00157-2 34022946 PMC8141191

[6] Ravindranath R, Bernstein IA, Fernandez KS, Ludwig CA, Wang SY. Social determinants of health and perceived barriers to care in diabetic retinopathy screening. JAMA Ophthalmol. 2023;141(12):1161-1171. doi:10.1001/jamaophthalmol.2023.5287 37971726 PMC10654926

[7] Woodcock EW. Barriers to and facilitators of automated patient self-scheduling for health care organizations: scoping review. J Med Internet Res. 2022;24(1):e28323. doi:10.2196/28323 35014968 PMC8790681

[8] Patel MP, Schettini P, O’Leary CP, Bosworth HB, Anderson JB, Shah KP. Closing the referral loop: an analysis of primary care referrals to specialists in a large health system. J Gen Intern Med. 2018;33(5):715-721. doi:10.1007/s11606-018-4392-z 29532299 PMC5910374

[9] Ezeonwu MC. Specialty-care access for community health clinic patients: processes and barriers. J Multidiscip Healthc. 2018;11:109-119. doi:10.2147/JMDH.S152594 29503559 PMC5826087

[10] Wang W, Lo ACY. Diabetic retinopathy: pathophysiology and treatments. Int J Mol Sci. 2018;19(6):1816. doi:10.3390/ijms19061816 29925789 PMC6032159

[11] Lundeen EA, Burke-Conte Z, Rein DB, . Prevalence of diabetic retinopathy in the US in 2021. JAMA Ophthalmol. 2023;141(8):747-754. doi:10.1001/jamaophthalmol.2023.2289 37318810 PMC10273133

[12] Gange WS, Lopez J, Xu BY, Lung K, Seabury SA, Toy BC. Incidence of proliferative diabetic retinopathy and other neovascular sequelae at 5 years following diagnosis of type 2 diabetes. Diabetes Care. 2021;44(11):2518-2526. doi:10.2337/dc21-0228 34475031 PMC8546279

[13] Kempen JH, O’Colmain BJ, Leske MC, ; Eye Diseases Prevalence Research Group. The prevalence of diabetic retinopathy among adults in the United States. Arch Ophthalmol. 2004;122(4):552-563. doi:10.1001/archopht.122.4.552 15078674

[14] Yan J, Li B, Chen Y, . Prevalence and predictors of developing vision-threatening diabetic retinopathy within the first three years of type 2 diabetes. Front Endocrinol (Lausanne). 2023;14:1305378. doi:10.3389/fendo.2023.1305378 38192422 PMC10773727

[15] Group TS; TODAY Study Group. Development and progression of diabetic retinopathy in adolescents and young adults with type 2 diabetes: results from the TODAY Study. Diabetes Care. 2021;45(5):1049-1055. doi:10.2337/dc21-1072 34531310 PMC9174974

[16] Lee R, Wong TY, Sabanayagam C. Epidemiology of diabetic retinopathy, diabetic macular edema and related vision loss. Eye Vis (Lond). 2015;2:17. doi:10.1186/s40662-015-0026-2 26605370 PMC4657234

[17] Patel D, Ananthakrishnan A, Lin T, Channa R, Liu TYA, Wolf RM. Social determinants of health and impact on screening, prevalence, and management of diabetic retinopathy in adults: a narrative review. J Clin Med. 2022;11(23):7120. doi:10.3390/jcm11237120 36498694 PMC9739502

[18] Mansberger SL, Gleitsmann K, Gardiner S, . Comparing the effectiveness of telemedicine and traditional surveillance in providing diabetic retinopathy screening examinations: a randomized controlled trial. Telemed J E Health. 2013;19(12):942-948. doi:10.1089/tmj.2012.0313 24102102 PMC3850428

[19] Lu Y, Serpas L, Genter P, Mehranbod C, Campa D, Ipp E. Disparities in diabetic retinopathy screening rates within minority populations: differences in reported screening rates among African American and Hispanic patients. Diabetes Care. 2016;39(3):e31-e32. doi:10.2337/dc15-2198 26721811

[20] Fairless E, Nwanyanwu K. Barriers to and facilitators of diabetic retinopathy screening utilization in a high-risk population. J Racial Ethn Health Disparities. 2019;6(6):1244-1249. doi:10.1007/s40615-019-00627-3 31463812 PMC6880869

[21] Lewis K. Improving patient compliance with diabetic retinopathy screening and treatment. Community Eye Health. 2015;28(92):68-69. 27418725 PMC4944097

[22] Ta AWA, Goh HL, Ang C, Koh LY, Poon K, Miller SM. Two Singapore public healthcare AI applications for national screening programs and other examples. Health Care Sci. 2022;1(2):41-57. doi:10.1002/hcs2.10 38938890 PMC11080681

[23] Scheetz J, Koca D, McGuinness M, . Real-world artificial intelligence-based opportunistic screening for diabetic retinopathy in endocrinology and indigenous healthcare settings in Australia. Sci Rep. 2021;11(1):15808. doi:10.1038/s41598-021-94178-5 34349130 PMC8339059

[24] Uy H, Fielding C, Hohlfeld A, . Diagnostic test accuracy of artificial intelligence in screening for referable diabetic retinopathy in real-world settings: a systematic review and meta-analysis. PLOS Glob Public Health. 2023;3(9):e0002160. doi:10.1371/journal.pgph.0002160 37729122 PMC10511145

[25] Goldstein J, Weitzman D, Lemerond M, Jones A. Determinants for scalable adoption of autonomous AI in the detection of diabetic eye disease in diverse practice types: key best practices learned through collection of real-world data. Front Digit Health. 2023;5:1004130. doi:10.3389/fdgth.2023.1004130 37274764 PMC10232822

[26] Goldstein J, Weitzman D, Abramoff MD. 69-OR: implementing autonomous AI in a federally qualified health center (FQHC) for the detection of diabetic retinopathy improves access to care: a pre–post comparison in the Southeastern U.S. Diabetes. 2022;71(suppl 1):69-OR. doi:10.2337/db22-69-OR

[27] Castro M, Bishop D, Weitzman D, Ramirez R. 57-OR: enhancing diabetic eye disease detection through autonomous artificial intelligence implementation in a federally qualified health center. Diabetes. 2024;73(suppl 1):57-OR. doi:10.2337/db24-57-OR

[28] National Association of Community Health Centers. America’s Health Centers: By the Numbers. Updated October 31, 2024. Accessed March 14, 2025. https://www.nachc.org/resource/americas-health-centers-by-the-numbers/

[29] Alowais SA, Alghamdi SS, Alsuhebany N, . Revolutionizing healthcare: the role of artificial intelligence in clinical practice. BMC Med Educ. 2023;23(1):689. doi:10.1186/s12909-023-04698-z 37740191 PMC10517477

[30] Matheny ME, Goldsack JC, Saria S, . Artificial intelligence in health and health care: priorities for action. Health Aff (Millwood). 2025;44(2):163-170. doi:10.1377/hlthaff.2024.01003 39841940

[31] Bajwa J, Munir U, Nori A, Williams B. Artificial intelligence in healthcare: transforming the practice of medicine. Future Healthc J. 2021;8(2):e188-e194. doi:10.7861/fhj.2021-0095 34286183 PMC8285156

[32] Davenport T, Kalakota R. The potential for artificial intelligence in healthcare. Future Healthc J. 2019;6(2):94-98. doi:10.7861/futurehosp.6-2-94 31363513 PMC6616181

[33] Feldstein AC, Glasgow RE. A practical, robust implementation and sustainability model (PRISM) for integrating research findings into practice. Jt Comm J Qual Patient Saf. 2008;34(4):228-243. doi:10.1016/S1553-7250(08)34030-6 18468362

[34] McCreight MS, Rabin BA, Glasgow RE, . Using the Practical, Robust Implementation and Sustainability Model (PRISM) to qualitatively assess multilevel contextual factors to help plan, implement, evaluate, and disseminate health services programs. Transl Behav Med. 2019;9(6):1002-1011. doi:10.1093/tbm/ibz085 31170296

[35] Bhaskaranand M, Ramachandra C, Bhat S, . The value of automated diabetic retinopathy screening with the EyeArt system: a study of more than 100,000 consecutive encounters from people with diabetes. Diabetes Technol Ther. 2019;21(11):635-643. doi:10.1089/dia.2019.0164 31335200 PMC6812728

[36] Ipp E, Liljenquist D, Bode B, ; EyeArt Study Group. Pivotal evaluation of an artificial intelligence system for autonomous detection of referrable and vision-threatening diabetic retinopathy. JAMA Netw Open. 2021;4(11):e2134254. doi:10.1001/jamanetworkopen.2021.34254 34779843 PMC8593763

[37] Lim JI, Regillo CD, Sadda SR, . Artificial intelligence detection of diabetic retinopathy: subgroup comparison of the EyeArt system with ophthalmologists’ dilated examinations. Ophthalmol Sci. 2022;3(1):100228. doi:10.1016/j.xops.2022.100228 36345378 PMC9636573

[38] Scanzera AC, Beversluis C, Potharazu AV, . Planning an artificial intelligence diabetic retinopathy screening program: a human-centered design approach. Front Med (Lausanne). 2023;10:1198228. doi:10.3389/fmed.2023.1198228 37484841 PMC10361413

[39] AlQudah AA, Al-Emran M, Shaalan K. Medical data integration using HL7 standards for patient’s early identification. PLoS One. 2021;16(12):e0262067. doi:10.1371/journal.pone.0262067 34972171 PMC8719694

[40] Eichelberg M, Aden T, Riesmeier J, Dogac A, Laleci GB. A survey and analysis of electronic healthcare record standards. ACM Comput Surv. 2005;37(4):277-315. doi:10.1145/1118890.1118891

[41] Strickland NH. PACS (picture archiving and communication systems): filmless radiology. Arch Dis Child. 2000;83(1):82-86. doi:10.1136/adc.83.1.82 10869010 PMC1718393

[42] Karawita C. Choice Maker - Random Picker. App. Updated June 14, 2020. Accessed September 6, 2025. https://apps.apple.com/us/app/choice-maker-random-picker/id1387970091

[43] Zulman DM, Rosland AM, Choi H, Langa KM, Heisler M. The influence of diabetes psychosocial attributes and self-management practices on change in diabetes status. Patient Educ Couns. 2012;87(1):74-80. doi:10.1016/j.pec.2011.07.013 21840149 PMC3229832

[44] Bursac Z, Gauss CH, Williams DK, Hosmer DW. Purposeful selection of variables in logistic regression. Source Code Biol Med. 2008;3:17. doi:10.1186/1751-0473-3-17 19087314 PMC2633005

[45] Liu TYA, Huang J, Channa R, . Autonomous artificial intelligence increases access and health equity in underserved populations with diabetes. Res Sq. Preprint posted online March 13, 2024. doi:10.21203/rs.3.rs-3979992/v1

[46] Crigger E, Reinbold K, Hanson C, Kao A, Blake K, Irons M. Trustworthy augmented intelligence in health care. J Med Syst. 2022;46(2):12. doi:10.1007/s10916-021-01790-z 35020064 PMC8755670

[47] Dawkins B, Renwick C, Ensor T, Shinkins B, Jayne D, Meads D. What factors affect patients’ ability to access healthcare? an overview of systematic reviews. Trop Med Int Health. 2021;26(10):1177-1188. doi:10.1111/tmi.13651 34219346

[48] Marshall EG, Miller L, Moritz LR. Challenges and impacts from wait times for specialist care identified by primary care providers: results from the MAAP study cross-sectional survey. Healthc Manage Forum. 2023;36(5):340-346. doi:10.1177/08404704231182671 37415463 PMC10448708

[49] Engineering a learning healthcare system: a look at the future: workshop summary. In: *Healthcare System Complexities, Impediments, and Failures*. Vol 3. National Academies Press; 2011. 21977540

[50] Maleki Varnosfaderani S, Forouzanfar M. The role of AI in hospitals and clinics: transforming healthcare in the 21st century. Bioengineering (Basel). 2024;11(4):337. doi:10.3390/bioengineering11040337 38671759 PMC11047988

[51] Seyed-Nezhad M, Ahmadi B, Akbari-Sari A. Factors affecting the successful implementation of the referral system: a scoping review. J Family Med Prim Care. 2021;10(12):4364-4375. doi:10.4103/jfmpc.jfmpc_514_21 35280649 PMC8884299

[52] Teisberg E, Wallace S, O’Hara S. Defining and implementing value-based health care: a strategic framework. Acad Med. 2020;95(5):682-685. doi:10.1097/ACM.0000000000003122 31833857 PMC7185050

