# Supplementary material for: Diabetic Retinopathy Screening Among Federally Qualified Health Center Patients Using Point-of-Care AI: DRES-POCAI: A Trial Protocol
Source: JAMA Netw Open. 2025 Oct 21;8(10):e2538114. doi: 10.1001/jamanetworkopen.2025.38114 (PMC12541539; doi:10.1001/jamanetworkopen.2025.38114)
Supplement: Supplement 1. — Trial Protocol and Statistical Analysis Plan [file jamanetwopen-e2538114-s001.pdf]

# Statistical Analysis Plan

**Study Title:** Diabetic Retinopathy Screening Point-of-Care Artificial Intelligence – DRES-POCAI

**Clinical Trial Registration**: ClinicalTrials.gov identifier: NCT06721351
https://clinicaltrials.gov/study/NCT06721351

**Sponsor**: Kaiser Permanente Research Institute for the Permanente Augmented Intelligence in Medicine and Healthcare Initiative (AIM-HI). Prime award funder is The Gordon and Betty Moore Foundation.

**Intervention**: Implementation of Artificial Intelligence tool for Diabetic Retinopathy Screening at point-of-care in a primary care setting.

**Statistical Analysis Plan Version:** Version 1.1

**Statistical Analysis Plan Date:** 13 August 2024

**Purpose:** The purpose of this analysis is to evaluate the impact of a point-of-care Artificial Intelligence Diabetic Retinopathy Screening tool (EyeArt [Eyenuk, Inc, Woodland Hills, CA]), on screening completion (primary outcome) and if changes in screening completion result in a corresponding increase in diagnosis of diabetic retinopathy (secondary outcome).

## STUDY DESIGN

**Design:** DRES-POCAI is a patient-level, multi-clinic, randomized controlled, open-label, parallel superiority trial.

**Study Aims and hypothesis: Aim 1** of the study is to refine and operationalize a multi-component AI diabetic retinopathy screening intervention (DRES-POCAI) and implementation plan with physicians, clinical staff, and patients from Federally Qualified Health Clinics in South and Central San Diego. **Aim 2** to evaluate the impact of DRES-POCAI implementation in a primary care setting among underserved patients with diabetes mellitus on diabetic retinopathy screening completion. We hypothesize (Hypothesis 2.1) that randomization to DRES-POCAI intervention will result in an increase in diabetic retinopathy screening completion and diabetic retinopathy detection when compared to usual care; (Hypothesis 2.2) that participants receiving DRES-POCAI intervention will be more likely to (a) complete referrals to the eye specialist, (b) establish early stages of diabetic retinopathy, and (c) be less likely to present diabetic retinopathy complications due to early treatment; and (Hypothesis 2.3) ) that participants receiving DRES-POCAI intervention will demonstrate greater (a) knowledge, (b) attitudes, (c) self-efficacy and (d) patient satisfaction regarding diabetic retinopathy, autonomous diabetic retinopathy screening, and other diabetes mellitus-related services and resources.

**Setting:** Selected clinics from the Federally Qualified Health Center of San Ysidro Health in San Diego County, California.

**Randomization:** Participants will be randomized using a simple block randomization design to ensure equal (1:1) allocation between intervention and standard of care arms by clinic site. Randomization will be performed using a statistical program or randomizer app (https://apps.apple.com/us/app/choice-maker-random-picker/id1387970091).

**Blinding:** Study staff and study data analysts will be unblinded due to the intervention activities.

**Study population:** Study participants will be active SYH patients receiving medical care in one of DRES-POCAI's research clinic sites and meet the following inclusion/exclusion criteria.

***Inclusion Criteria***

- A diagnosis of diabetes myelitis
- Age >22 years
- No retinal screening during the previous 11 months
- A medical visit scheduled during the intervention period
- Ability to read and understand English or Spanish

***Exclusion Criteria***

- A prior diagnosis of diabetic retinopathy, macular edema, or retinal vascular occlusion
- Persistent visual impairment in one or both eyes
- A history of ocular injections, retinal laser treatment, or intraocular surgery (excluding cataract surgery)
- Pregnancy
- A diagnosis of mental or degenerative disease that precluded self-consent

***Outcome Measures:***

***Primary outcome***

- Completion of retinal screen (AI-DRS-System or screening by an ophthalmologist) within 3 months of enrollment and medical visit.

***Secondary outcomes***

- Diabetic retinopathy diagnosis as determined by EyeArt or ophthalmologist
  - EyeArt-System results: normal, mtmDR, vtDR, or ungradable
  - Eye specialist referral clinical classification, using ICD-10 codes: E10.9, E11.9, E10.329, E11.329; E10.339, E11.339; E10.321, E11.321, E10.331, E11.331, E10.349, E11.349, E10.341, E11.341, E10.359, E11.359, E10.351, E11.351.
- Completion of retinal screen (AI-DRS-System or screening by an ophthalmologist) within 6 months of enrollment

***Exploratory outcomes***

- Patient education/knowledge outcomes as measured at baseline and 6 months
- Comparison of EyeArt diagnostic results to DR diagnosis by an eye specialist when evaluated by both methods for each individual eye.

***Covariates****:* clinic site, age, sex, prior screening history, clinical risk factors, social determinates of health, barriers to care, and diabetes knowledge and self-efficacy.

## STATISTICAL METHODS

**Sample Size and Power Calculations:** Based on historical data from participating SYH clinics, we determined that approximately 59% of patients completed diabetic retinopathy screening, we powered our study to detect an increase of at least 10% in retinopathy screening completion (59% in the standard of care group to 69% in the intervention group), and assuming an α of 0.05 (significance) and a β of 0.8 (power), we estimated that the target enrollment to detect this difference is 722 participants (361 per arm) using a two independent sample Pearson $\chi 2$ test. Accounting for up to 15% attrition from consent to study intervention (participants who agree to participate and consent via phone, and either do not keep their appointment or who at the clinic appointment decide to withdraw from the study), we estimated as many as 848 patients (424 per arm) would need to be enrolled to ensure adequate sample size. To assess secondary outcomes, we determined that among the patients who underwent retinal screening within the previous 12 months at the participating SYH clinics, 11% were diagnosed with diabetic retinopathy; therefore, based on a screening uptake increase of 10%, a corresponding 16% increase in diabetic retinopathy diagnosis is anticipated in the intervention arm. However, if after the initial refinement

phase of the project, it is determined that either a smaller or larger percent of the target population is currently completing DR screening, we will adjust the study enrollment accordingly, to ensure we will have sufficient statistical power to evaluate the anticipated 10% increase in screening uptake.

**Statistical significance and transformation of variables**: All statistical tests will be two-sided unless defined otherwise. Statistical significance will be set at p-value <0.05. Where distributional assumptions are necessary for the correct evaluation of statistical results, appropriate description, diagnostic evaluation, and (if required) transformation of variables will be carried out. In the event the parametric tests indicated are unable to be used, non-parametric versions will be used.

**Reporting conventions**: P-values will be reported with two significant figures, but no longer than 4 decimal places (e.g. p<0.0001, p=0.0001, p=0.0098, p=0.098, p=0.98). Distribution estimates such as mean, standard deviation, median, and quartiles will be reported to 3 decimal places. Parameters estimates such as regression coefficients, confidence intervals and hazard ratios will be reported to two significant digits (e.g. 1.23, 95% CI 0.69, 1.77).

**Missing data**: Primary analysis will be carried out without imputation of outcome variables. Multiple deletion methods will be used to handle missing covariate data and will be dependent on the pattern of missingness, e.g. if entire surveys are missing, if missingness is driven by specific items or response groups in the survey, or if the data is missing completely at random. We have allowed for a 15% dropout/withdrawal rate between enrollment and intervention and thus can use listwise deletion of up to 15% of participants and still maintain adequate power for analysis.

**Data summaries**: Continuous data will be summarized using medians and interquartile range (IQR), mean (SD), or geometric mean (95% CI). Categorical data will be summarized with frequency and percentages (based on non-missing sample size) using all observed levels. Data will be listed and sorted by arm and visit number where appropriate. Summary tables will be structured with a column for each arm and will be annotated with the total population size relevant to that table/arm, including any missing observations.

**Analysis Populations:** All consented participants who were randomized will be considered the intent-to-treat (ITT) population. Participants in the ITT population who were not excluded after enrollment will be considered the per-protocol population (PP). The ITT population will be used as the primary population for the analysis. Subgroups will be analyzed using both ITT and PP populations.

**General Approach**: All analyses will be performed at a patient level. Baseline demographics and characteristics will be presented by treatment arm using standard descriptive statistics. No hypothesis testing will be used to compare the distribution of baseline covariates between intervention and standard of care treatment arms.

The primary analysis will compare diabetic retinopathy screening completion between the intervention group and the standard of care group. Logistic regression will be used to estimate intervention efficacy using treatment arm as the main exposure variable and the odds ratio (OR) including 95 % CI will be presented. Hosmer and Lememshow's approach to purposeful selection of variables will be used for final model selection.

Secondary analysis will compare diabetic retinopathy screening results between treatment arms using the same logistic regression approach as the primary analysis. Missing treatment results will be classified in this analysis and no diagnosis.

**Exploratory analysis**: Exploratory analyses will be performed to assess relationships between screening completion, screening outcomes, social determinates of health, and clinical risk factors. Changes in patient education/knowledge outcomes will be assessed using difference in

differences analysis. These analyses will be used to identify items that are most strongly related to barriers and/or compliance, and be sued to refine and guide future implementation strategies.

Additionally, where both EyeArt and ICD-10 codes are available, results will be compared and shared with EyeArt, however these results will not generalizable as only results from participants in the intervention group who received ungradable or abnormal EyeArt results and who were examined by an eye specialist and had results returned to the SYH will be available for comparison.

## DATA MANAGEMENT

**Methods for data collection:** Data will be captured using electronic record forms in REDCap. EMR data will be extracted and entered into REDCap forms by study personnel and participants will complete survey questionnaires using study provided electronic tablets.

**Data quality and standards**: The quality of data collection and data entry will be maximized through training of staff in both administration of the intervention and the standardized questionnaire administration. Study staff will be trained in the data collection and will be expected to demonstrate competence before extracting data from the EHR.

Verified and validated data will be stored on secure, fault-tolerant network servers at San Ysidro Health. Data will be backed-up on a continuous basis on a secure off-site server and on encrypted standalone hard drives. Once the data validation phase has been completed, the database will be locked and transferred to a statistical programmer who will do further syntax-driven consistency checks and syntax-driven data cleaning.

The study programmer will have access to the original copies of the source data. He/she will then prepare the database for data analysis by the statistician by generating the final variables for data analysis, such as the construction of the composite endpoints. The final cleaned database will be available with a corresponding data dictionary.

**Safety analysis and data monitoring**: No formal safety endpoints will be analyzed. However, all documented adverse events will be recorded and presented to the Data Safety Monitoring Committee (DSMC) and in the final report detailing the date, description, resolution, follow-up, and outcome.

To ensure participant safety and study integrity, the DSMC will meet quarterly to review recruitment progress, data quality, and protocol adherence, and to advise on managing any special circumstances.

**Interim Analyses:** No formal interim analysis for efficacy is planned.

**Statistical analysis software**: Data will be cleaned using MS Excel and analyzed using STATA.

## REPORTING AND DISSEMINATION

**Reporting guidelines:** We will follow the Consolidated Standards of Reporting Trials (CONSORT) statement guidelines (https://jamanetwork.com/journals/jamasurgery/article-abstract/2778467) for reporting and dissemination of trial results.

**Participant disposition and flow chart**: The CONSORT table or flowchart will include the number of subjects screened, enrolled, randomized, and subsequently found to be screen failures.

**Metadata standards and data documentation**: The full study protocol, supporting documents including the data management Standard Operating Procedures (SOPs) and the full individual participant level database will be made available upon request after study findings have been published and approval by the SYH FQHC research review committee.

**Data preservation and safety standards**: The research data will be stored long-term in the original electronic format, in a database that contains all research data excluding identifiable participant data. Linking file for identifiable data will be stored separately. The data will be preserved for minimum of 7 years after study completion.

# Diabetic Retinopathy Screening Point-of-Care Artificial Intelligence *DRES-POCAI*: AI - Clinical Intervention at San Ysidro Health, Aim 2

**Protocol Number: 810204**

**National Clinical Trial (NCT) Identified Number: Pending**

**Principal Investigator: Fatima Muñoz**

**Co-Principal Investigator: Sonia Tucker, San Ysidro Health**

**UCSD PI: Nicole Stadnick**

**IND/IDE – N/A**

**Funded by:** Gordon and Betty Moore Foundation, through Kaiser Permanente Division of Research

**Version Number: v.1.2**

**February 19, 2025**

**Summary of Changes from Previous Version:**

| Affected Section(s) | Summary of Revisions Made | Rationale |
|---|---|---|
| | | |
| | | |

## Table of Contents


1 PROTOCOL SUMMARY ........ 1
1.1 Synopsis ........ 1
1.2 Schema ........ 3
1.3 Schedule of Activities (SoA) ........ 4
2.0 INTRODUCTION ........ 5
2.1 Study Rationale ........ 5
2.2 Background ........ 5
2.3 Risk/Benefit Assessment ........ 7
2.3.1 Known Potential Risks ........ 7
2.3.2 Known Potential Benefits ........ 8
2.3.3 Assessment of Potential Risks and Benefits ........ 8
3 OBJECTIVES AND ENDPOINTS ........ 10
4 STUDY DESIGN ........ 11
4.1 Overall Design ........ 11
4.2 Scientific Rationale for Study Design ........ 12
4.3 Justification for Dose ........ 13
4.4 End of Study Definition ........ 13
5 STUDY POPULATION ........ 14
5.1 Inclusion Criteria ........ 14
5.2 Exclusion Criteria ........ 14
5.3 Lifestyle Considerations ........ 15
5.4 Screen Failures ........ 15
6 STUDY INTERVENTION ........ 15
6.1 Study Intervention(s) Administration ........ 15
6.1.1 Study Intervention Description ........ 15
6.1.2 DOSING and Administration ........ 16
5.2 Preparation/Handling/Storage/Accountability ........ 16
5.2.1 Acquisition and accountability ........ 16
5.2.2 Formulation, Appearance, Packaging, and Labeling ........ 16
5.2.3 Product Storage and Stability ........ 16
5.2.4 Preparation ........ 16
6.3 Measures to Minimize Bias: Randomization and Blinding ........ 16
6.4 Study Intervention Compliance ........ 17
6.5 Concomitant Therapy ........ 18
6.5.1 Rescue Medicine ........ 18
7 STUDY INTERVENTION DISCONTINUATION AND PARTICIPANT DISCONTINUATION/WITHDRAWAL ........ 18
7.1 Discontinuation of Study Intervention ........ 18
7.2 Participant Discontinuation/Withdrawal from the Study ........ 19
7.3 Lost to Follow-Up ........ 19
8 STUDY ASSESSMENTS AND PROCEDURES ........ 20
8.1 EFFICACY Assessments ........ 20
8.2 Safety and Other Assessments ........ 22
8.3 Adverse Events and Serious Adverse Events ........ 24
8.3.1 Definition of Adverse Events (AE) ........ 24
8.3.2 Definition of Serious Adverse Events (SAE) ........ 25
8.3.3 Classification of an Adverse Event ........ 25

8.3.4. Time Period and Frequency for Event Assessment and Follow-Up ........ 26
8.3.5 Adverse Event Reporting ........ 26
8.3.6 Serious Adverse Event Reporting ........ 27
8.3.7 REPORTING EVENTS TO PARTICIPANTS ........ 27
8.3.8 Events of Special Interest ........ 27
8.3.9 Reporting of Pregnancy ........ 27
8.4 Unanticipated Problems ........ 28
8.4.1 Definition of Unanticipated Problems (UP) ........ 28
8.4.2 Unanticipated Problem Reporting ........ 28
8.4.3 Reporting Unanticipated Problems to Participants ........ 28
9 STATISTICAL CONSIDERATIONS ........ 29
9.1 Statistical Hypotheses ........ 29
9.2 Sample Size Determination ........ 29
9.3 Populations for Analyses ........ 30
9.4 Statistical Analyses ........ 30
9.4.1 General Approach ........ 30
9.4.2 Analysis of the Primary Efficacy Endpoint(s) ........ 30
9.4.3 Analysis of the Secondary Endpoint(s) ........ 31
9.4.4 Safety Analyses ........ 32
9.4.5 Baseline Descriptive Statistics ........ 32
9.4.6 Planned Interim Analyses ........ 32
9.4.7 Sub-Group Analyses ........ 32
9.4.8 Tabulation of Individual participant Data ........ 32
9.4.9 Exploratory Analyses ........ 32
10 SUPPORTING DOCUMENTATION AND OPERATIONAL CONSIDERATIONS ........ 33
10.1 Regulatory, Ethical, and Study Oversight Considerations ........ 33
10.1.1 Study Discontinuation and Closure ........ 33
10.1.2 Confidentiality and Privacy ........ 33
10.1.3 Future Use of Stored Specimens and Data ........ 34
10.1.4 Key Roles and Study Governance ........ 35
10.1.5 Safety Oversight ........ 35
10.1.6 Clinical Monitoring ........ 35
10.1.7 Data Handling and Record Keeping ........ 37
10.1.8 Protocol Deviations ........ 38
10.2 Additional Considerations ........ 39
10.3 Abbreviations ........ 40
10.4 Protocol Amendment History ........ 41
11 REFERENCES ........ 42

# 1 PROTOCOL SUMMARY

*Leave blank. Text should be included under the relevant subheadings below.*

## 1.1 SYNOPSIS

**Title:** Diabetic Retinopathy Screening Point-of-Care Artificial Intelligence - DRES-POCAI: AI - Clinical Intervention at San Ysidro Health (SYHealth).

**Study Description:** This study is intended to address unmet medical needs in diabetic eye care in a community health center setting by enhancing and modifying existing clinical practices with the integration of point-of-care (POC) artificial intelligence (AI) technology for Diabetic Retinopathy (DR) screening. The study will optimize, implement, and test the impact of a multicomponent intervention that includes: 1) autonomous DR screening, a fast and non-invasive retinal exam into the primary care settings with 2) integration of the results into the EHR and 3) health education/care coordination support (e.g., patient education).

**Objectives:** *Primary Objective (Clinical):* Evaluate the implementation and effectiveness of a multicomponent AI clinical intervention on DR screenings rate, early stages of DR detection, and referrals to the specialist for follow up on abnormal results.

*Secondary Objectives:* Evaluate the implementation and effectiveness of a multicomponent AI clinical intervention on DR knowledge, attitudes, self-efficacy, and patient satisfaction.

**Endpoints:** *Primary Endpoint (Clinical):* DR screening status, stages of DR detection, and referrals to the specialist for follow up on abnormal results.

*Secondary Endpoints:* DR patient engagement, intention to engage in future DR screenings.

**Study Population:** Participants will be active SYHealth patients 22 years of age or older with diabetes mellitus (DM) who have not had a retinal exam in the last 11 months, and have a medical visit scheduled during the intervention period and are able to read and understand either English or Spanish in order to provide informed consent and complete study surveys. Exclusion criteria: 1) have a prior diagnosis of DR, macular edema, or retinal vascular occlusion; 2) have persistent visual Impairment in one or both eyes; 3) history of ocular injections, laser treatment of the retina, or intraocular surgery (excluding cataract surgery); 4) pregnant women; and 5) diagnosis of mental or degenerative disease that prevents self-consent for the study.

The study will recruit a cohort of 848 adults from SYHealth-Chula Vista (SYH-CV) and SYHealth-King Chavez (SYH-KC) clinics.

**Phase:** Not applicable

| | |
|---|---|
| **Description of Sites/Facilities Enrolling Participants:** | Enrollment sites for the study will include two primary care clinics within SYHealth's service network, SYH-CV and SYH-KC. The two selected clinics are in the South and Central regions respectively. These sites serve two of SYHealth's primary service areas with the highest prevalence of DM. SYH-CV is the medical home to more than 24,000 patients (~13% with a diagnosis of DM). The clinic is in the second largest city in San Diego County, with approximately 75% of its residents identifying as Hispanic/Latino, and 13% of the population living below 100% of the federal poverty level (FPL). SYH-KC serves as a medical home for more than 9,000 patients (~14% with a diagnosis of DM). The clinic is situated in a community with primarily racial and ethnic minority residents of color (SYHealth population ~60% Hispanic/Latino and 16% Black), with 14% of the residents in this community living below the 100% FPL and disproportionately impacted by social determinants of health (SDoH). SYHealth's quality metrics of these two clinics reflect high prevalence of DM, and conversely, poor disease management. For example, DR screening (46%) is significantly low and demonstrates a need for further improvement of clinical practices/workflows. |
| **Description of Study Intervention:** | Study staff will generate a data query including patients with DM. A biweekly eligibility report will be generated from the electronic health record (EHR), listing patients who meet the eligibility criteria with scheduled medical appointments. Trained study staff contact potential participants (by phone or in person) and will explain the study , invite them to be part of the research study and, if they are interested, the staff will schedule a study visit to complete consent and surveys on site. Once the potential participants arrive to their study visit appointment, they will complete the consent process. During the study visit participants will be randomized into either the intervention or usual care group. A simple block randomization design will ensure equal allocations between intervention and usual care arms by clinic and assign participants to equal (1:1) allocation. For the usual care group, DR screenings with the optometrist will be scheduled according to SYHealth's protocols. The optometrist will provide the results during the visit, discuss the appropriate follow-up and document results in the medical record. For the intervention group, DR screenings will be conducted using the EyeArt® AI system (Eyenuk, Inc.) in-clinic prior to the medical visit (same day if the visit is 'in-person', or within the previous week if the medical visit is via telehealth). Results will be available in the Eyenuk web portal and can be opened directly within SYHealth's EHR. DR Screening uptake will be extracted from the EHR along with DR diagnosis, clinical, and demographic data. Increases in participant knowledge, attitudes, and self-efficacy will be collected using pre- and post- surveys. Measures will also include participant satisfaction with autonomous DR screening, and other DM-related services and resources. |
| **Study Duration:** | *2 years* |
| **Participant Duration:** | *1 year* |

## 1.2 SCHEMA

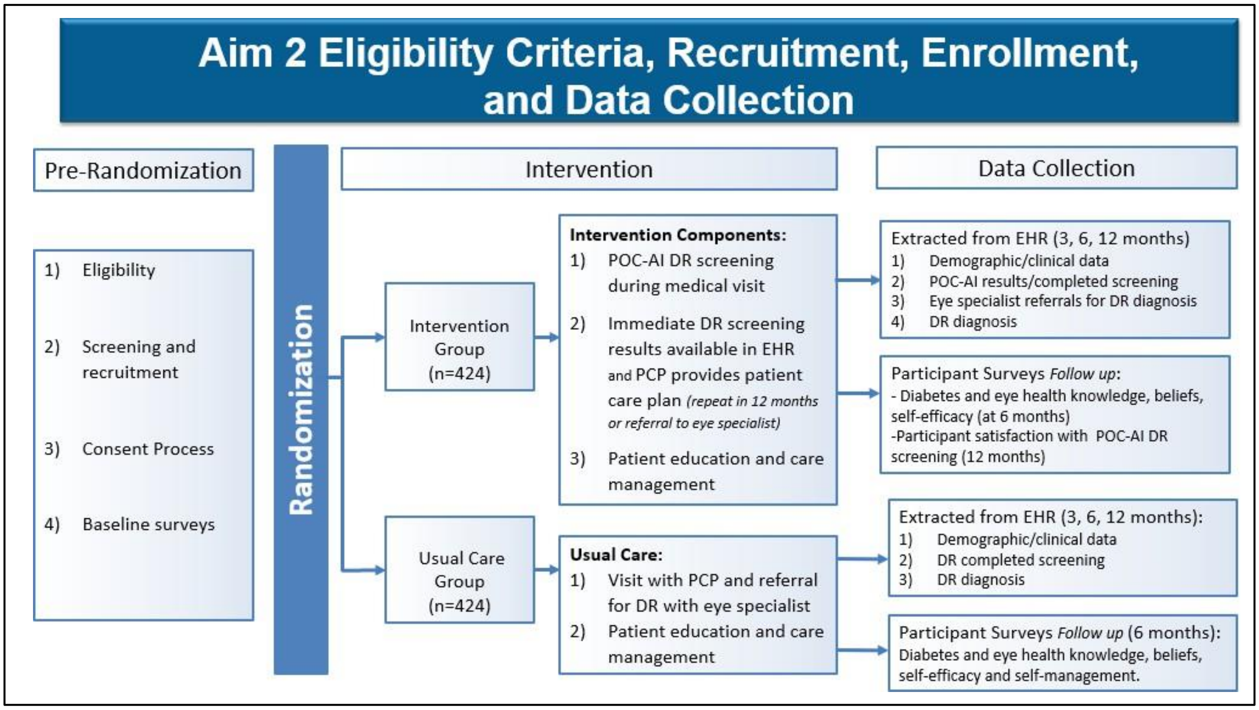
Aim 2 Eligibility Criteria, Recruitment, Enrollment, and Data Collection
Pre-Randomization
Randomization
Intervention
Data Collection
1) Eligibility
2) Screening and recruitment
3) Consent Process
4) Baseline surveys
Intervention Group (n=424)
Usual Care Group (n=424)
Intervention Components:
1) POC-AI DR screening during medical visit
2) Immediate DR screening results available in EHR and PCP provides patient care plan (repeat in 12 months or referral to eye specialist)
3) Patient education and care management
Usual Care:
1) Visit with PCP and referral for DR with eye specialist
2) Patient education and care management
Extracted from EHR (3, 6, 12 months)
1) Demographic/clinical data
2) POC-AI results/completed screening
3) Eye specialist referrals for DR diagnosis
4) DR diagnosis
Participant Surveys Follow up:
- Diabetes and eye health knowledge, beliefs, self-efficacy (at 6 months)
-Participant satisfaction with POC-AI DR screening (12 months)
Extracted from EHR (3, 6, 12 months):
1) Demographic/clinical data
2) DR completed screening
3) DR diagnosis
Participant Surveys Follow up (6 months):
Diabetes and eye health knowledge, beliefs, self-efficacy and self-management.

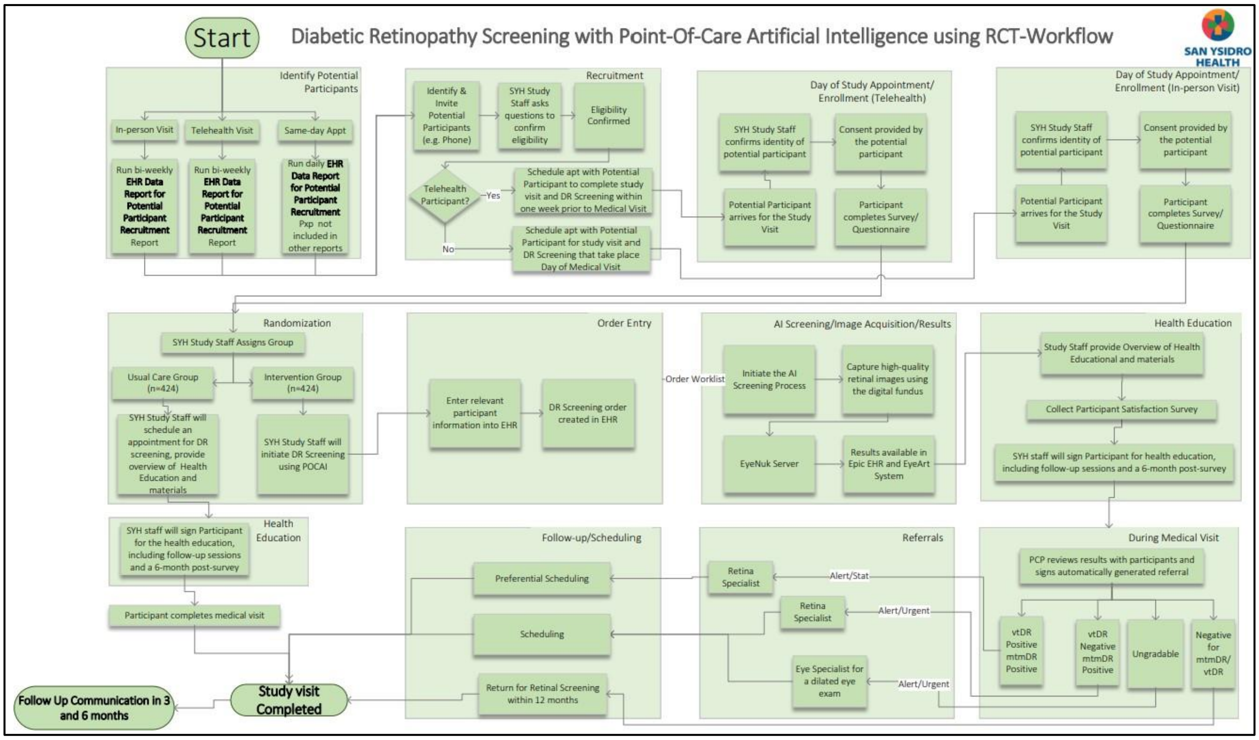
Start
Diabetic Retinopathy Screening with Point-Of-Care Artificial Intelligence using RCT-Workflow
SAN YSIDRO HEALTH
Identify Potential Participants
In-person Visit
Telehealth Visit
Same-day Appt
Run bi-weekly EHR Data Report for Potential Participant Recruitment Report
Run bi-weekly EHR Data Report for Potential Participant Recruitment Report
Run daily EHR Data Report for Potential Participant Recruitment Pxp not included in other reports
Recruitment
Identify & Invite Potential Participants (e.g. Phone)
SYH Study Staff asks questions to confirm eligibility
Eligibility Confirmed
Telehealth Participant?
Yes
No
Schedule apt with Potential Participant to complete study visit and DR Screening within one week prior to Medical Visit
Schedule apt with Potential Participant for study visit and DR Screening that take place Day of Medical Visit
Day of Study Appointment/ Enrollment (Telehealth)
SYH Study Staff confirms identity of potential participant
Consent provided by the potential participant
Potential Participant arrives for the Study Visit
Participant completes Survey/ Questionnaire
Day of Study Appointment/ Enrollment (In-person Visit)
SYH Study Staff confirms identity of potential participant
Consent provided by the potential participant
Potential Participant arrives for the Study Visit
Participant completes Survey/ Questionnaire
Randomization
SYH Study Staff Assigns Group
Usual Care Group (n=424)
Intervention Group (n=424)
SYH Study Staff will schedule an appointment for DR screening, provide overview of Health Education and materials
SYH Study Staff will initiate DR Screening using POCAI
Order Entry
Enter relevant participant information into EHR
DR Screening order created in EHR
Order Worklist
AI Screening/Image Acquisition/Results
Initiate the AI Screening Process
Capture high-quality retinal images using the digital fundus
EyeNuk Server
Results available in Epic EHR and EyeArt System
Health Education
Study Staff provide Overview of Health Educational and materials
Collect Participant Satisfaction Survey
SYH staff will sign Participant for health education, including follow-up sessions and a 6-month post-survey
Health Education
SYH staff will sign Participant for the health education, including follow-up sessions and a 6-month post-survey
Participant completes medical visit
Follow-up/Scheduling
Preferential Scheduling
Scheduling
Return for Retinal Screening within 12 months
Referrals
Retina Specialist
Retina Specialist
Eye Specialist for a dilated eye exam
Alert/Stat
Alert/Urgent
Alert/Urgent
During Medical Visit
PCP reviews results with participants and signs automatically generated referral
vtDR Positive mtmDR Positive
vtDR Negative mtmDR Positive
Ungradable
Negative for mtmDR/ vtDR
Study visit Completed
Follow Up Communication in 3 and 6 months

## 1.3 SCHEDULE OF ACTIVITIES (SOA)

***This is an example and could be modified as appropriate or replaced.***

| **Procedures** | Pre-recruitment | Recruitment | Enrollment/Study Visit (Baseline) | 6 months | 12 months | 18 months | 24 months |
|---|---|---|---|---|---|---|---|
| Eligibility review and Aim 1 co-design | X | | | | | | |
| Eligibility Screening | X | X | | | | | |
| Schedule Study Visit | X | X | | | | | |
| Informed Consent | | | X | | | | |
| Demographics and baseline survey | | | X | | | | |
| Randomization | | | X | | | | |
| Administer study intervention: DRES-POCAI | | | X | | | | |
| Participant education overview and Engagement resources | | | X | | | | |
| Routine Medical Visit | | | X | | | | |
| Follow up health education sessions (3 sessions), 6-months survey after intervention | | | | X | | | |
| Administer follow-up DR screening (12-months from first screening) and participant satisfaction survey on DRES-POCAI | | | | | X | | |
| Follow up with Eye Specialist and referrals for DRES-POCAI abnormal results | | | X-------------------------X | | | | |
| Study Completion | | | | X -------------------- X | | | |

## 2.0 INTRODUCTION

*Leave blank. Text should be included under the relevant subheadings below.*

### 2.1 STUDY RATIONALE

San Ysidro Health (SYHealth) is a Federally Qualified Health Center (FQHC) serving San Diego County's medically underserved minority communities that are low-income, underinsured, with high rates of diabetes mellitus (DM) and low rates of diabetic retinopathy (DR) screening. SYHealth has identified an innovative method to address unmet medical needs in diabetic eye care by enhancing and modifying existing clinical practices. SYHealth will integrate point-of-care (POC) artificial intelligence (AI) technology for DR screening. The DRES-POCAI intervention will be developed collaboratively by an interdisciplinary team of clinical, AI technology, and research experts to integrate POC AI into SYHealth's electronic health records (EHR) and increase access to DR screening within the primary care setting. This will improve quality performance measures, facilitate clinical decision-making process, accelerate timely identification of DR, linkage to DM guideline-concordant care, and educate/engage patients, leading to improved overall management of eye health.

### 2.2 BACKGROUND

**Health disparities in preventive care impact DR screening rates in an FQHC setting.** DR is the leading cause of blindness among working-aged adults, affecting approximately 26.3% of people with DM[1,2] due to individuals not receiving timely vision-saving interventions. DR is mostly asymptomatic even at advanced stages of the disease, making it a significant medical and public health challenge for improving patient clinical outcomes[2]. Clinical studies have demonstrated that timely treatment can reduce the risk of vision impairment, making blindness preventable,[2,3] however, only 35% to 60% of people with DM in the US receive annual DR screening.[4] This is consistent for medically underserved ethnic minority FQHC Medicaid patients, only 39% with DM visit an eye care provider annually, and only 29% of their uninsured counterparts access these services. This is likely due to the unique individual and structural barriers affecting FQHC patient populations. Patient-level barriers to DR screening include lack of knowledge and education about DR[5], perceived risk, language, work schedules, accessibility of DR screening sites, and insurance.[6]

The probability of an individual accessing eye care every year decreases among those with a lower income and educational level.[5] Structural level barriers include transportation, financial hardships, long wait times,[7,3] lack of access to eye care providers, and gaps in current primary care workflows. Hispanic, African American, and Asian communities are known to have higher rates of DM, which makes screening in these communities even more critical.[1] A population-based study of Latinos 40 years and older residing in Southern California found that 36% had an eye care visit in the last 12 months, only 19% had a dilated eye exam, and less than 60% had a dilated eye exam in their lifetime.[5]

**DR Screening Practices – Usual Care Model in FQHCs.** Comprehensive dilated eye exams can detect, stage, monitor DR (including mild, moderate, and severe non-proliferative and proliferative DR) and help clinicians provide early intervention for DR to prevent vision loss[5,8]. However, DR screening is not a standard of care in primary care clinics. In areas with limited eye care providers, people may delay routine eye exams or treatment until the onset of eyesight-threatening problems[9]. American Diabetes Association (ADA), American Academy of Ophthalmology (AAO), and American Optometric Association (AOA) all recommend that individuals with type 2 DM have a dilate comprehensive eye exam to screen for DR at

the time of diagnosis and follow-ups as advised by their eye care provider.[1,2 -10]Although the current standard of care for routine eye exams is available, patients find it inconvenient. From a clinical and implementation perspective, this model is inefficient, with limited capacity to meet the growing demand for sight-preserving DR screening within a community-based clinical setting. [11,12] Innovative clinical approaches are necessary to establish early DR diagnosis and treatment, especially among underserved populations. These efforts should include a strategy to integrate POC DR screening in the primary care workflow and increase DR education to ensure adherence.

**Clinical Impact of Proposed Use Case.** Using EyeArt® AI system (Eyenuk, Inc.), an FDA-cleared AI algorithm for DR screening, [19,20] SYHealth proposes a RCT to assess the effectiveness of the AI system in promoting and completing eye exams among patients with DM. ***Refer to Appendix 2 for a description of the device***. The ADA, AAO, AOA, and Center for Disease Control and Prevention (CDC) recommends that people with DM undergo annual eye exams and maintain a hemoglobin A1c (HbA1c) <7% to prevent the development of DR and vision loss. Aligned with these recommendations, the proposed study will focus on adult patients >22 years with a diagnosis of DM due for annual DR screening. SYHealth proposes to implement the DRES - POCAI in an FQHC environment that will 1) improve accessibility for individuals who may face challenges in accessing specialized eye care facilities by integrating POC AI within primary care settings; 2) integrate POC AI with EHR to facilitate clinical decision-making process and patient care; 3) empower primary care providers to offer comprehensive care for individuals with DM; 4) accelerate timely identification of DR, and linkage to DM guideline-concordant care; 5) increase access to DR screening and quality performance rates; and 6) educate and engage patients, improving overall management of eye health. This research study presents a sustainable, promising practice to address the growing need for DR prevention, early diagnosis, and treatment that can be disseminated in primary care settings. Furthermore, this study offers important methodological and content-related advances to improve knowledge about implementing real-world AI/ML capabilities in a community health center that addresses gaps in the healthcare decision-making process.

**AI Algorithm and Performance*:*** The EyeArt AI system (EyeArt) has a clinically aligned framework and is trained on 375,000 images and validated on over 250,000 images.[32] The system requires a digital fundus camera to capture two 45° field of view images, one centered on the optic disc and one centered on the macula. These images may be taken without dilation. EyeArt has been validated in a prospective, multicenter, pivotal clinical trial including 942 individuals with DM, where EyeArt demonstrated high accuracy against the Early Treatment Diabetic Retinopathy Study (ETDRS) reference standard clinical reference standard (mtmDR sensitivity: 96%, specificity: 88%, and vtDR sensitivity: 97%, specificity: 90%) by providing conclusive reports for >97% of eyes with reference standard while not requiring dilation for most.[20] In a retrospective study of more than 100,000 consecutive encounters with people with DM, EyeArt also achieved a performance of 91.3% sensitivity and 91.1% specificity in detecting mtmDR.[23] A subgroup analysis of participants in this clinical trial demonstrated that the sensitivity against the ETDRS reference standard for detection of mtmDR was significantly greater with EyeArt than with either an ophthalmologist dilated examination. Therefore, the FDA-cleared EyeArt is suitable for the prospective evaluation proposed in this application. The AI algorithms in EyeArt will be static (fixed) during the proposed evaluation process and will not be retrained. Therefore, no data requirements are associated with retraining the AI algorithms in EyeArt. Based on the existing EyeArt AI algorithm outcomes, SYHealth will implement a patient-centered model for its population of focus. In all four outcomes (mtmDR positive, vtDR positive), (mtmDR positive, vtDR negative), (mtmDR negative, vtDR negative), and ungradable, SYHealth's model will integrate a trained SYHealth clinical staff that will ensure patient care to proper follow up based on the EyeArt AI system results and as indicated by the primary care

provider. Preliminary Studies that guide the proposed AI System for autonomous DR screening are listed in **Appendix 3*.***

**Project relation to existing organizational priorities and initiatives.** In 2016, SYHealth adopted the patient-centered medical home (PCMH) model across its clinics. This approach seeks to deliver high-quality, cost-effective primary care in a culturally inclusive manner by supporting a team-based approach that puts patients and their families at the center of service delivery. As the PCMH model has been enhanced and expanded, SYHealth's current efforts include a focused approach to improving population health, patient care experience (including quality and satisfaction), and reducing health care disparities specifically for patients at high-risk for or living with DM. This proposed study seeks to identify and integrate clinical best practices for DM care by a) enhancing the delivery of services by the care team and b) maximizing the use of technology within the primary care setting, e.g., enhancing diagnostic-decision making via AI technology, EHR data, and leveraging patient-level data to inform interventions and/or outreach efforts. The implementation of an innovative autonomous AI-DR screening will align with and support SYHealth's organizational priorities and initiatives to improve DR screening, early DR diagnosis, to reduce the risk of severe vision loss, and improve clinical outcomes in SYHealth patients with DM. Findings from SYHealth's research intervention will help identify best practices for scalability and capacity building, such as data privacy, algorithm accuracy, and ongoing evaluation to optimize the impact of Artificial Intelligence/Machine Learning (AI/ML) technology for diagnostic decision-making in real-world settings.

## 2.3 RISK/BENEFIT ASSESSMENT

*Leave blank. Text should be included under the relevant subheadings below.*

### 2.3.1 KNOWN POTENTIAL RISKS

<u>Participation in the study may involve potential risks or discomforts.</u>

- **Some survey questions might be uncomfortable.** Participants do not have to answer anything that makes them feel uncomfortable. Participants may choose to skip questions or stop participating at any time.
- **Retinal screening could be uncomfortable.** Participants in the intervention group will complete a retinal screening that may cause some brief discomfort (light flashes, eye strain). Participants will not be dilated for this type of screening and the possible discomforts are momentary. It is very safe, and the study team will be there to assist participants through any discomfort. Participants may also choose to stop at any time.
- **Abnormal results are possible.** Retinal screening is part of routine medical care. If the study team finds something that needs attention, the participants doctor will discuss the appropriate care (e.g., referral to the eye specialist) with the participants. It will be the participants choice whether to proceed with additional tests and/or treatments to evaluate what was observed. The participant and/or payer will be responsible for any associated costs.
- **Risks of Loss of Confidential Information:** Measures will be taken to keep information private, however there is always a small risk that unauthorized personnel can get access to the study data. To reduce this risk, the research team will use a unique identification number or code instead of the participants name to protect their privacy. The study data will be protected with a password and only the study researchers and staff will have access to the files.

- **New information.** If the study team learns of important findings during the study that could affect the participants' health or their decision to participate, they will be informed in a timely manner. There might also be unknown risks – these would likely be minor and temporary.
- **Possible Unknown Risks:** In addition, unknown risks may be temporary, mild, and last only while the individual is actively participating in the research study. Risks also could be serious, long-lasting, and may even cause death. Participants will be informed of any new findings that might affect their health, welfare or might impact their willingness to continue participating in the research study.

### 2.3.2 KNOWN POTENTIAL BENEFITS

Participation may result in early detection of eye problems from diabetes, helping participants receive timely advice and support for better diabetes management. The possible benefits regardless of research group assignment, include participants will obtain helpful information about eye health and diabetes self-management that will facilitate DR screening adherence; navigation support to schedule follow-up appointments, as needed. In addition, participants assigned to the intervention group will also receive a retinal screening without dilation using the EyeArt® AI system; the DR screening will be completed before their medical provider visits. The results will be available immediately after the screening, allowing participants to learn about and discuss their eye health with their care provider.

Participation will provide researchers with information to help others with diabetes and may also improve eye care within the primary care setting Within the context of health disparities and structural barriers, there are many factors that limit access to timely DR screening, especially in underserved populations. Existing care models are inefficient and inconvenient for patients. The integration POC-AI of DR screening within primary care workflows has the potential to improve accessibility to DR screening, facilitate early DR diagnosis and treatment, and empower primary care providers to offer comprehensive care for individuals with DM.

### 2.3.3 ASSESSMENT OF POTENTIAL RISKS AND BENEFITS

The risks of participation in the study outweigh the value of the information to be gained. The research team anticipates the study results and learnings will reduce the risk of severe vision loss and improve clinical outcomes for individuals who choose to participate. The selected POC- AI for DR screening is FDA-approved and uses a camera system to obtain retina images without the need for dilation. Thus, implementation of the screening poses no more than minimal risk to the subject.

There is a potential risk that patients who receive a negative (i.e., normal) DR screening result may feel comfortable and assume they no longer need complete their recommended regular eye exam. This could lead to overlooking other critical conditions such as glaucoma, cataracts or deteriorating visual acuity and the need for vision correction lenses. The health education and care coordination component of the intervention will provide participants with materials and education emphasizing the importance of maintaining routine visual care. Health educators will also emphasize that POC-AI screening specifically targets diabetic retinopathy (DR), therefore cannot replace the need for routine comprehensive eye exams. Although other personal special benefits from participating in the study are not guaranteed, the research study team anticipates participation may result in early detection of eye problems associated with DR. This early detection can provide individuals with timely health education and support to enhance their diabetes self-management practices and improve clinical outcomes.

*Participant Experience:* Efforts will be made to minimize potential discomfort associated with participating in any aspect of this research. Sharing experiences accessing health care and receiving health information may prove to be uncomfortable for participants. To mitigate this risk the research team will hire interviewers who are bicultural and bilingual or multilingual to ensure that consenting, surveys and screenings are conducted in a culturally appropriate and respectful manner. Participants will be informed that deciding not to participate in the study will not impact their ability to receive services from SYH.

*Informed Consent:* The informed consent will be conducted in a private office to ensure confidentiality and minimize distractions, allowing participants to focus on the information being provided and ask questions freely. The research study will have a designated office at each clinic site (Chula Vista and King-Chavez) where participants will complete the consent, surveys and DR screening. The staff will ensure that the setting is comfortable, and they create an environment with the participants to open communication, respect the participant's autonomy and privacy, and allows for the thorough discussion of the study details and participant questions. The study staff will inform participants that they may discontinue their participation at any point in time with no impact on their clinical treatment. There are no known or anticipated serious health or psychological risks of participating in this study.

*Surveys/Questionnaires:* Some of the questions we ask may make participants feel uncomfortable. Study staff will emphasize that they are not obligated to answer any questions that make them uncomfortable, and that they can skip any question or stop participation at any time.

*Diabetic Retinopathy Screenings*: Participants assigned to the POC-AI group may experience discomfort during the DR screening, such as light flashes or eye strain. It's important to note that these potential discomforts are minimal and momentary. Additionally, participants will not undergo dilation for the POC-AI DR screening, only participant in usual care group. The DR screening is part of a participant’s medical care. While abnormal results may cause concern or stress, participants should understand that these results do not constitute a final diagnosis. The study staff will explain that the results of the screening are not the final diagnosis, and the results should be reviewed by their medical provider. The medical provider will review the results with the participant and discuss any recommended treatment plan, such as a referral to an eye specialist. Ultimately, it will be the participant's choice whether to proceed with additional tests or treatments to evaluate what was observed.

*Participant Information/Data:* We will take measures to keep the information collected private, however, there is also a risk that participants’ information could be released to an unauthorized party. To minimize this risk, we will use a unique identification number or code on any information we collect, and we will keep a link between the code and identity in a different location. The study database and data sources will be password protected and only the researchers and study staff will have access to the files. Personally identifiable data will not appear in any research data. As is true for all SYHealth and UC San Diego personnel, any person(s) working on this research project will under extensive Human Research Protection and HIPAA training, including topics around the e potential issues regarding the maintenance and protection of confidentiality (e.g., sending confidential material to a community printer, using names while conducting interviews, etc.).

The information to be gained about the impact of clinical intervention holds significant value, which outweighs the minimal risks associated with the research study. Furthermore, the research team is well-equipped to implement measures to mitigate and protect against the identified risks.

# 3 OBJECTIVES AND ENDPOINTS

| OBJECTIVES | ENDPOINTS | JUSTIFICATION FOR ENDPOINTS |
|---|---|---|
| Primary | | |
| Evaluate the implementation and effectiveness of a multicomponent AI clinical intervention on DR screenings, early stages of DR detection, and referrals to the specialist for follow up on abnormal results. | 1. Completion of DR screening. Data will be obtained through EHR at the time of the enrollment (baseline).<br><br>2. Results of the DR screening: for usual care specific diagnosis; for intervention group defined as normal, mild-to-moderate DR (mtmDR), visual threatening DR (vtDR) or ungradable. Data will be obtained through EHR at the time of the enrollment (baseline) and over the duration of the study.<br><br>3. Referral to specialist completion status and final diagnosis. Data will be obtained through EHR over the duration of the study. | *To evaluate the effectiveness of the clinical intervention will be tested using a RCT (Aim 2), with the "intervention group", which is characterized by 1) conducting autonomous DR screening system (H2.1) with 2) integration of the results into the EHR (H2.2).The "usual care" group represents the existing primary care model, which involves medical visits, referral to the specialist for a DR screening, and follow-up.* In addition, to evaluating the impact and efficiency of the POC-AI System we will also collect AI system data, number of orders submitted, screenings completed (images), number of ungradable and time of attempts, time the patient spent in front of the camera. |
| Secondary | | |
| Evaluate the implementation and effectiveness of a multicomponent AI clinical intervention on diabetes and DR knowledge, attitudes, self-efficacy, and patient satisfaction. | 1. A 15 min-survey will be conducted at the baseline and 6 months. The survey will assess patients' knowledge, attitudes about DR (e.g., "Do you think your DM can make you blind"),[41] self-efficacy and ("I am confident that I can: take care of my eyes")[42]. Participants will be asked about their comfort and trust in the use and results of the novel POC-AI screening that does not involve | We hypothesize that our intervention strategies will exert their impact on implementation and clinical outcomes through diabetes and DR knowledge, attitudes, self-efficacy, and patient satisfaction (**H2.3**). |

| OBJECTIVES | ENDPOINTS | JUSTIFICATION FOR ENDPOINTS |
|---|---|---|
| | seeing a provider, and their overall satisfaction with the intervention.<br>2. Participants will be asked their consent to obtain additional EHR data, such as sex, date of birth, SES (e.g., education, insurance), marital status, ethnicity, etc. In addition, measures of access and barriers (SDoH) to health care, personal and family health history related to DM, current and past smoking, and alcohol intake and clinical data (e.g., anthropometry, blood pressure, HbA1c, lipids, nephropathy exam, etc.). | *Additional data is required to delineate participant characteristics, individual-level barriers, and additional clinical information. This data is necessary for evaluating the effectiveness of the intervention across subgroups/categories by including a risk group classification variable in our models (to determine if there are differences in outcomes by group).* |
| Tertiary/Exploratory | | |
| Not applicable | | |

# 4 STUDY DESIGN

*Leave blank. Text should be included under the relevant subheadings below.*

## 4.1 OVERALL DESIGN

To evaluate the implementation and effectiveness of a multicomponent AI clinical intervention using a patient-level randomized controlled trial (RCT) for underserved patients with DM receiving medical care in FQHC clinics in South and Central San Diego.

- Compared to controls, at both follow-up points, participants receiving DRES-POCAI will report: **Hypothesis 2.1** greater screening rates of DR; expect a 10% improvement in DR adherence rates; **Hypothesis 2.2** will be more likely to (a) complete referrals to the eye specialist, (b) establish early stages of DR, and (c) less likely to present DR complications due to early treatment; and **Hypothesis 2.3** greater (a) knowledge, (b) attitudes, (c) self-efficacy and (d) patient satisfaction about DR, autonomous DR screening, and other DM-related services and resources.

- *Phase of the trial, if applicable*
  - Not applicable
- *A description of the type/design of trial to be conducted (e.g., randomized, placebo-controlled, double-blinded, parallel design, open-label, dose escalation, dose-ranging, adaptive, cluster randomized, group sequential, multi-regional, superiority or non-inferiority design)*
  - This is a randomized controlled trial (RCT). Patients will be randomized into either the intervention or usual care arm when they arrive for their appointment. A simple block randomization design will ensure equal allocations between intervention and usual care arms by clinic and assign participants to equal (1:1) allocation between arms.

Randomization will be performed using a statistical program (random number generator) to allocate study assignments.

- *A description of methods to be used to minimize bias*
  - Drs. Muñoz and Stadnick will work closely to manage any potential missing data issues, to measure and correct potential biases, including administrative and fiscal oversight.
- *Dose escalation or dose-ranging details should be contained in* ***Section 6.1.2, Dosing and Administration***
  - Not applicable
- *The number of study groups/arms and study intervention duration*
  - There will be two study arms as participants will be randomized to the intervention (autonomous DR screening or Standard-of-Care). The intervention is for a period of 1 year.
- *Indicate if single site or multi-site*
  - Single site working with SYHealth. Locations are the King-Chavez and Chula Vista clinics.
- *Name of study intervention(s)*
  - Diabetic Retinal Screening Point of Care Artificial Intelligence by using a special camera and a computer system called *EyeArt®.*
- *Note if interim analysis is planned and refer to details in* ***Section 9.4.6, Planned Interim Analysis***
  - Not applicable
- *Note if the study includes any stratifications and if so, identify the stratification planned (e.g. sex, race/ethnicity, age, dose) and refer to details* ***in Section 9.4.7, Sub-Group Analyses***
  - Not applicable
- *Name of sub-studies, if any, included in this protocol*
  - Not applicable

## 4.2 SCIENTIFIC RATIONALE FOR STUDY DESIGN

The DRES-POCAI study is using a randomized controlled, open-label, parallel superiority trial. The benefit of this trial is that each participant will be randomized to two intervention options, allowing researchers to compare the relative effectiveness of multiple intervention options. The DRES-POCAI study employs a randomized controlled, open-label, parallel superiority trial design. This design is well-suited to achieve the study's aims for several reasons:

**Randomization**: Random assignment of participants to intervention and control groups minimizes confounding variables. This ensures that any observed differences in outcomes are likely due to the intervention itself and not pre-existing differences between the groups. Participants of the DRES-POCAI study will be randomized into two groups: usual care (receive a retinal screening by an optometrist) or intervention group (receive a retinal screening using a special camera and EyeArt® system, *an autonomous AI-based DR screening*). The randomization will occur after consenting and completing the surveys, prior to conducting the DR screening process (for those randomized to the intervention group).

**Open-label Design**: The open-label nature is appropriate given the practical realities of implementing a multi-component intervention with integrated technology. Blinding participants and providers to the autonomous DR screening is logistically difficult. The intervention's immediate results and potential impact on the workflow necessitate an open-label approach.

**Parallel Design**: The parallel design allows for a clear, side-by-side comparison of outcomes between the intervention and usual care groups over time. This establishes the efficacy of the multicomponent approach. Also, this approach streamlines research analysis and clearly compares outcomes within the defined study period.

**Superiority Evaluation**: The primary objective is to determine if the multi-component DRES-POCAI intervention is superior to usual care in increasing DR screenings, early detection, referrals, and patient knowledge/self-efficacy. A superiority trial directly establishes whether the intervention offers significant benefits over the current model.

Overall, the DRES-POCAI study employs a rigorous and well-justified design that balances scientific rigor with ethical considerations and the practical realities of implementation in FQHC settings. The selected study design provides a rigorous and pragmatic framework for evaluating the DRES-POCAI intervention and supports the potential to generate meaningful evidence for improving diabetic retinopathy care in underserved populations.

## 4.3 JUSTIFICATION FOR DOSE

**Not applicable**

## 4.4 END OF STUDY DEFINITION

The end of the study is defined as the completion of the last study-related activity at the close of the 2-year study. Participants of the DRES-POCAI study are considered to have completed the study if they have completed all the phases of the study, including the last scheduled DR screening shown in the **Schedule of Activities (SoA), Section 1.3.** A DRES-POCAI participant's individual study period will be considered complete upon the occurrence of the following, whichever comes latest:

**Completion of Baseline Visit:** During the first study visit, participants will complete consent, fill out a brief survey about diabetes care and eye health. At this time, the participant will also be randomized and assigned to the usual care or intervention group.
**Completion of Diabetic Retinopathy Screening:** The intervention group will complete the DR screening using a special camera and the AI system (EyeArt®), the same day of the study visit. The usual care group will complete the DR screening with an eye care provider on a different day and at a different location. The study staff will facilitate this process for participants in the usual care group by assisting them in scheduling appointments for their routine retinal screening. The study staff will provide an overview of the health education sessions, provide engagement and educational materials and schedule the follow-up health education visits with all participants. This step will ensure the completion of this important component.

**Participant Satisfaction Survey (Intervention Group Only)**: Participants will complete the end-of-study satisfaction survey.
**Medical visit:** Both study groups will complete a medical visit after the screening. If the medical visit is in-person, then participants will complete it the same day as the screening. If the medical visit is via telehealth, then participants will complete it on a different day after the screening.

**Completion of Health Education Program:** Attendance at all scheduled health education sessions (3 sessions within the 6 months after the study visit) along with completion of any associated post-program evaluation surveys.

**One-Year Follow-Up Screening (Intervention Group Only):** Participants who had a negative initial DR screening will complete a follow-up screening one year after their initial enrollment and the participant satisfaction survey.

**The overall DRES-POCAI study will be considered complete** when participants' recruitment, all follow-up activities (including screenings and surveys) have been finalized, and the collected data has been cleaned, verified, and is ready for analysis. Participants assigned to the usual care group are anticipated to complete their participation within a six-month timeframe, and participants assigned to the intervention group are anticipated to complete the study's research activities at the 12 months from the initial study visit.

Participants may end their participation at any point of the intervention without completing the research activities in the following circumstances:

**Withdrawal**: Refers to participants who voluntarily decide to discontinue their involvement in the study before its completion.

**Lost to Follow-up**: Refers when participants fail to attend scheduled follow-up screenings, and /or are unreachable despite a defined number of contact attempts as defined in the protocols.

# 5 STUDY POPULATION

*Leave blank. Text should be included under the relevant subheadings below.*

## 5.1 INCLUSION CRITERIA

This study targets active SYHealth patients with diabetes mellitus (DM) who have not had a retinal exam in the last 11 months. The study will recruit and randomize a cohort of 848 adults (424 per arm) from SYHealth-Chula Vista and SYHealth-King-Chavez clinic sites. In order to be eligible to participate in this study, an individual must meet all of the following criteria:

1. Provision of signed and dated informed consent form.
2. Stated willingness to comply with all study procedures and availability for the duration of the study.
3. Established and active patient of SYHealth-CV and KC (having a medical appointment in the last 18 months).
4. Person aged 22 and older.
5. Established diagnosis of DM.
6. Medical appointment(s) (in-person or telehealth) scheduled during the intervention period.
7. Has not completed a dilated eye exam or retinal exam in the last 11months.
8. Be able to read, understand, and speak English or Spanish at a level sufficient to consent to participation, comprehend educational materials and sessions, and complete surveys.

## 5.2 EXCLUSION CRITERIA

The study will exclude individuals who present any of the following conditions, as there is no benefit to screening or insufficient data to prove safety and/or benefit (e.g., pregnancy). Additionally, selected criteria align with EyeArt® contraindications based on the FDA clearance.

1. Prior diagnosis of eye problems including retinopathy, blocked blood vessels, macular edema, or retinal vascular occlusion.
2. Persistent visual impairment in one or both eyes.
3. History of ocular injections, laser treatment of the retina, or intraocular surgery (excluding cataract surgery).
4. Individuals with contraindication for fundus photography (for example, have symptomatic light hypersensitivity).
5. Pregnant women.
6. Diagnosis of mental or degenerative disease that prevents self-consent for the study.

## 5.3 LIFESTYLE CONSIDERATIONS

For the DRES-POCAi study, no lifestyle considerations will be part of the eligibility screening process.

## 5.4 SCREEN FAILURES

The eligibility screening will be completed before consenting potential participants and randomization **(Study schema 1.2)**. For individuals who do not meet the eligibility criteria or are unable to participate in the DRES-POCAI research study, the study staff will express gratitude for their time and interest in the study. Individuals will be given contact information to schedule their routine retinal screening appointments (SYHealth's Quality department). Additionally, the staff will provide handouts about diabetes and eye health to encourage these individuals to maintain their diabetes care and management with their SYHealth primary care provider.

The study does not anticipate screen failures as defined as "participants who are consented to participate in the clinical trial, who do not meet one or more criteria required for participation".

# 6 STUDY INTERVENTION

## 6.1 STUDY INTERVENTION(S) ADMINISTRATION

*Leave Blank. Text should be included under the relevant subheadings below.*

### 6.1.1 STUDY INTERVENTION DESCRIPTION

The purpose of the DRES-POCAI research study is to improve eye care by using a special camera and a computer system called EyeArt® to make diabetic eye screenings faster and more accessible. Eligible participants that choose to participate will complete a survey about diabetes management and eye health. They will also be randomized and assigned to one of the two study groups: usual care (receive a retinal screening by an eye care provider) or intervention group (receive a retinal screening using a special camera and EyeArt). The study visit (baseline) is expected to be completed in a period of ~60 minutes.

<u>During the first study visit, participants will</u>:

1. Complete informed consent
2. Fill out a survey on diabetes, eye health, and barriers to retinal screening completion.
3. Undergo randomization and be assigned to a study group: usual care or intervention.
4. Undergo DR Screening. *Intervention group* participants will complete the DR screening using a special camera and the artificial intelligence (AI) system (EyeArt®) on the same day of the study

visit. Participants will also complete a satisfaction survey related to the use of EyeArt system to complete their DR screening.

***Usual care group* participants will complete the DR screening with an eye care provider on a different day and at a different location. The study staff will assist in scheduling appointments for the DR screening to ensure the completion of this important component.

5. Health Education Overview and Engagement materials: at the end of the study visit, the study staff will provide participants with an overview of the health education sessions. Staff will also ensure participants have scheduled any/all follow-ups. Participants will receive educational materials about diabetes care and management, eye health, etc.

*Complete medical visit*: Participants will complete a medical visit on the same day as the study visit (for in-person visits). If the medical visit is via telehealth, then participants will complete it on a different day after the screening.

Follow up contact with participants: After the first (baseline) study visit, all participants will participate in one 20-minute educational session and complete a short survey within 6 months of the baseline. Upon the completion of the second health education session and the 6-month follow-up survey, participants will receive a $25 gift card.

For participants in the intervention group who had a negative initial DR screening, they will be asked to complete their follow-up DR screening one year after the initial enrollment.
Both arms will be encouraged to follow up with their eye care provider for comprehensive eye exams.

#### 6.1.2 DOSING AND ADMINISTRATION

**Not applicable**

### 5.2 PREPARATION/HANDLING/STORAGE/ACCOUNTABILITY

#### 5.2.1 ACQUISITION AND ACCOUNTABILITY

**Not applicable**

#### 5.2.2 FORMULATION, APPEARANCE, PACKAGING, AND LABELING

**Not applicable**

#### 5.2.3 PRODUCT STORAGE AND STABILITY

**Not applicable**

#### 5.2.4 PREPARATION

**Not applicable**

### 6.3 MEASURES TO MINIMIZE BIAS: RANDOMIZATION AND BLINDING

To minimize study intervention bias and variability, the study is using a randomized controlled, open-label, parallel superiority trial. By randomizing, research participants have an equal opportunity of being

selected into the intervention or control group of the study. Using a computer generated (randomizer application on a tablet or touchscreen device) random number assignments will be given. This process will help generate comparable groups, thereby minimizing the effects of variables other than the variable under evaluation. Randomization will allocate participants into either the Intervention group, which will utilize a Point-of-Care Topcon® non-mydriatic retinal camera connected to the EyeArt® AI software for DR screening, or the control group, which will receive usual care screening, in a 1:1 ratio, respectively. This method aims to minimize bias and ensure that the differences observed between the two groups can be attributed to the intervention itself rather than other external factors.

The research study staff – Research Assistants (RA) will be responsible for the randomization process. The study team will implement strict procedures to ensure that the allocation sequence is conducted during the participant group assignment with the RAs. No allocation will be determined before a participant arrives. This process will prevent selection bias and ensures that study personnel cannot influence group assignment based on participant characteristics or interest. The RAs will press a designated button on the application to initiate the random assignment, the assigned group will display clearly on the screen for the participant to either the intervention group or the control group.

To mitigate the bias due to the lack of blindness, objective outcomes such as DR screening rates and referrals, as well as validated patient surveys regarding knowledge and self-efficacy, will be used. Furthermore, the research team will implement strict measures to minimize open-label bias. This includes:

a) Establishing standardized procedures for data collection, assessment, and outcome measurement to mitigate the influence of bias on study results. This ensures consistency and reliability across all study groups.

b) Implementing robust data monitoring processes overseen by the Data Safety and Monitoring Committee (DSMC) to safeguard the integrity and validity of study findings.

c) Employing statistical methods to adjust for potential biases, such as sensitivity analysis to address differences between treatment groups. These measures collectively strengthen the reliability and credibility of the study outcomes.

Regarding control group expectations, participants in the usual care group may be less motivated to seek external eye exams knowing the immediate screening results are not available during the study. The study will emphasize the importance of comprehensive eye exams in both groups to mitigate this. In addition, both usual care and intervention groups will receive health education materials and care coordination support, this is anticipated to increase engagement for participants in the usual care group.

By implementing these measures, the study team will minimize bias in randomization and enhance the validity and reliability of study results, addressing the absence of blinding.

## 6.4 STUDY INTERVENTION COMPLIANCE

The study team has established a protocol for ensuring study intervention compliance and quality assurance that involves coordinated efforts of study leadership and team members with the implementation of different strategies listed below:

1. Role Assignments:
    a. Program Manager (PM): Oversees the quality framework, including protocol development and report management. Provides weekly reports on potential participants and addresses technical needs related to data collection and management.

   b. Clinical Trials Coordinator (CTC): Assists the PM in quality control, staff training, and daily checks.
   c. Research Assistant (RA): Responsible for maintaining protocol adherence, ensuring data integrity, and participating in ongoing training.
   d. Data Analyst (DA): Generates weekly, biweekly or monthly reports on participant engagement, appointment outcomes, randomization and demographic data, while ensuring data accuracy.
2. Protocol Adherence: The PM and CTC will collaborate to develop and maintain the study protocol, ensuring that all staff members understand and adhere to the established procedures. RAs will be responsible for actively monitoring participant interactions and ensuring compliance with the intervention protocol during study visits, especially during randomization and DR screenings. Weekly and biweekly reports generated by the DA will provide insights into protocol adherence and help identify any deviations that need to be addressed.
3. Quality Control and Training: The PM and CTC will oversee staff training sessions to ensure that all team members are equipped with the necessary knowledge and skills to implement the intervention protocol effectively and maintain integrity of the intervention. Daily checks will be conducted by the CTC to help identify any issues or challenges related to the protocol adherence and provide opportunities for immediate corrective action. The PM will communicate with Eyenuk to obtain the appropriate training and quality control follow-ups for the operation of the Autonomous AI DR screening system (camera and EyeArt computer system).
4. Data Management: The DA will work closely with the PM and CTC to ensure the accuracy and integrity of study data, conducting regular audits and checks to identify and correct any discrepancies. The DA will also work closely with IT team to provide support with data management efforts by providing technical assistance and addressing any issues related to data collection and storage.
5. Collaboration and Accountability: All study staff members are collectively responsible for maintaining protocol adherence, data quality, and scientific integrity. The study leadership will have weekly meetings and close communication with all team members to facilitate a cohesive effort towards the success of the study. In addition, the PM will conduct monthly QA/QC competency check list to ensure the team performance is following all study protocols and procedures and are up to date with the possible changes or adjustments.

## 6.5 CONCOMITANT THERAPY

**Not applicable**

### 6.5.1 RESCUE MEDICINE

**Not applicable**

# 7 STUDY INTERVENTION DISCONTINUATION AND PARTICIPANT DISCONTINUATION/WITHDRAWAL

*Leave blank. Text should be included under the relevant subheadings below.*

## 7.1 DISCONTINUATION OF STUDY INTERVENTION

Participation in the DRES-POCAI research study is voluntary, allowing participants to decline participation or withdraw at any time without needing to provide a reason or facing any penalties. Participants may

withdraw voluntarily at any time by contacting one of the study staff members or PI. The PI will remove a participant from the study if they find this to be in their best interest. If a participant discontinues their study participation, they will be considered withdrawn from the study. Any data collected from these individuals will be retained up until their point of withdrawal. Additionally, the study may also discontinue participation for reasons such as retrospective identification of ineligibility.

## 7.2 PARTICIPANT DISCONTINUATION/WITHDRAWAL FROM THE STUDY

Participants may withdraw from the study at any time upon request. This action will lead to the removal of their identifying data from research data, ensuring privacy and compliance. Withdrawn participants will also be removed from active participant logs and include them in withdrawn participant logs, maintaining accurate study records. This process is to respect participant autonomy while ensuring data integrity and privacy.

The investigator may discontinue or withdraw a participant from the study for the following reasons:

- Significant study intervention non-compliance
- If any clinical adverse event (AE), or other medical condition or situation such that continued participation in the study would not be in the participant's best interest.
- If the participant meets an exclusion criterion (either newly developed or not previously recognized) that precludes further study participation, including pregnancy.

The reason for participant discontinuation or withdrawal from the study will be also recorded on the participant's Case Report Form (CRF). Research participants who sign the informed consent form and are randomized but do not receive the study intervention may be replaced. Research participants who sign the informed consent form, and are randomized and receive the study intervention, and subsequently withdraw, or are withdrawn or discontinued from the study, will not be replaced but the outcome of the participant's progress will be reported as part of the study progress report.

## 7.3 LOST TO FOLLOW-UP

Participant retention will be supported by several factors. First, participants will be recruited who have an existing appointment scheduled with their primary care provider at the participating clinic sites. Second, regularly scheduled reminders through phone calls and message notifications (text or email) will keep participants engaged in the study and in close communication with the research team. A participant will be considered lost to follow-up if they fail to complete a survey and are unable to be contacted by the study site staff after consenting and completing the baseline study visit.

The following actions must be taken if a participant fails to return to the clinic for a required study visit or fails to complete their health education sessions (by phone, virtual or in person):

- The study staff will attempt to contact the participant and reschedule the missed visit within a 1 month window period and counsel the participant on the importance of maintaining the assigned visit schedule and ascertain if the participant wishes to and/or should continue in the study.
- Before a participant is deemed lost to follow-up, the study staff will make every effort to regain contact with the participant, where possible, 3 telephone calls, 3 texts or email, and, if necessary, a letter to the participant's known mailing address or local equivalent methods the participant

agreed to be contacted. These contact attempts will be documented in the participant's medical record and CRF.

- Should the participant continue to be unreachable, participant will be considered to have withdrawn from the study with a primary reason of lost to follow-up.

# 8 STUDY ASSESSMENTS AND PROCEDURES

*Leave it blank. Text should be included under the relevant subheadings below.*

## 8.1 EFFICACY ASSESSMENTS

The DRES-POCAI study will evaluate the implementation and effectiveness of the multicomponent AI clinical intervention using a patient-level randomized control trial among underserved patients with diabetes receiving medical care in FQHC clinics in South and Central San Diego. The study procedures and evaluations will support the determination of efficacy, as per the primary and secondary objectives that are outlined in this section.

To evaluate the implementation and effectiveness of the multicomponent AI clinical intervention DRES-POCAI (hypothesis (section 4.2)) analyses will be performed at the patient level. Initially SYHealth will compare the intervention and control groups to determine if there is a significant (H2.1) increase in retinal screening uptake in the intervention group (primary outcome) and a corresponding (H2.2) increased diagnosis of DR (secondary outcome).

For both the primary and secondary outcomes SYHealth will compare the intervention group to the control group using two-sample t tests for continuous variables and $\chi^2$ test will be used for binary and categorical variables. If any significant imbalance is detected for any baseline characteristics, a sensitivity analysis will be performed, and the unbalanced characteristics will be included in the regression outcome models. For the survey exploratory analyses will be performed to assess relationships between each item in the (H2.3) education questionnaire and the screening behavior (i.e., the proportion of patients with completed DR screen). These analyses will be used to identify items most strongly related to barriers and/or compliance, and thus, help refine the tool and guide future implementation strategies.

*Selection of eligible potential participants*. The sequence of the intervention activities that should occur during the recruitment, screening and any decisions points regarding participant eligibility are represented in the workflow diagram below and eligibility screening questions (***SOPs Recruitment and Eligibility Screening*****).**

1. **Identification of potential participants**. The study coordinator will generate biweekly reports from the EHR to identify SYHealth active patients with upcoming and immediately following completed appointments. Additionally, this will only include patients recommended for follow-up medical visits within the next three months according to the standard of care for diabetes. Reports will be generated every Thursday, one week before their appointment with the primary care provider. The EHR lists will include information to confirm eligibility criteria according to the study protocol and contact information to facilitate initial communication with potential participants.
2. **Potential participant outreach**. Study staff will verify eligibility by comparing key information on the report with the information in the individual's EHR, e.g., DM diagnosis, established & active SYHealth patient, and the last documented date of a DR screening before calling the potential participant. Then, staff will introduce the research study, using a standard low-literacy script they will provide an overview of the study, invite them and obtain verbal consent. Contact outcomes: *a)* Individual agrees

to participate, staff will document in the study data collection system that verbal consent was obtained and will ask the individual to respond a few questions to confirm identity and eligibility **(see SOP Eligibility Screening and Identity Verification)**. Then, staff will schedule the appointment for the study visit. *b)* individual declines participation, study staff will provide information about DR screening and encourage the individual to complete their eye exam as soon as possible. Individuals will receive detailed instructions of how to schedule their DR screening appointment with the eye specialist. *c)* unsuccessful contact attempt, staff will make up to three initial contact attempts documenting the outcome.

3. **Eligibility screening to potential participants**. This process will involve the verification of eligibility of potential participants who meet specific criteria for the DRES-POCAI. Utilizing EHR-generated lists, telephone communication, and in-person reviews, the process ensures a thorough assessment of potential participants. For potential participants who have been pre-screened through EHR reports and phone calls, their in-person visit to the clinic/study site is the next step to validate their eligibility and formalize their participation in the study. Staff will use the eligibility screening questionnaire **(SOPs Appendix Eligibility Screening Questionnaire)** to guide the in-person eligibility screening process and confirm participation of the interested individual.

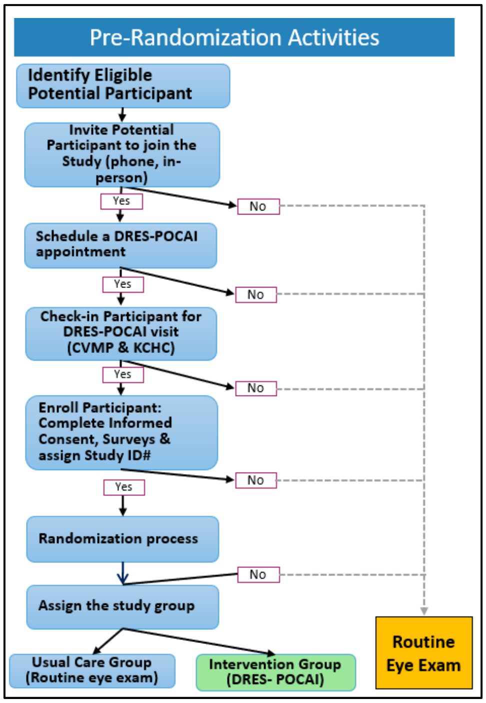


<u>*Enrollment of Participants*</u>. The sequence of the intervention activities that should occur during the enrollment process includes informed consent, data collection of participants, and randomization process (***SOPs Informed Consent and Randomization***).

1. **Informed Consent**. The informed consent will be conducted in a private office at each clinic study site (Chula Vista and King-Chavez) to ensure confidentiality and minimize distractions, enabling participants to focus on provided information and ask questions freely. Potential participants will have the flexibility to select their preferred method to complete the informed consent, electronic or printed version. Study staff will provide support for technical, literacy, and clarification needed during the consent process to maintain consistency across enrollment sites and uphold each potential participant's decision-making autonomy. Consent materials will be available in English and Spanish, verbal translation of any aspect of the informed consent process without official translation will not be allowed **(Appendices 4 and 5).**
2. **Randomization**. Participants of the DRES-POCAI study will be randomized into two groups: usual care (receive a retinal screening by an optometrist) or intervention group (receive a retinal screening using a special camera and EyeArt® system, an autonomous AI-based DR screening). The randomization will occur after consenting and completing the surveys, prior to conducting the DR screening process. The study coordinator will guide participants through the randomization process using a "*Randomizer*" app on a tablet or touchscreen device. The participant will press a designated button on the app to initiate random assignment to either the Intervention or Control group. The assigned group will display clearly on the screen for the participant to see.

_Intervention components and procedures_. The sequence of the study activities that should occur during the intervention includes the health education sessions, administration of surveys and DR screening (***SOPs Surveys, Diabetic Retinopathy Screening and Participant Education***).

1. **Administration of surveys.** Baseline and follow-up surveys will encompass the baseline and 6-month follow up surveys, including the following domains, a) diabetes knowledge and beliefs, b) diabetes self-efficacy and c) diabetes self-management. In addition, participants of the intervention group will be asked to also complete participant satisfaction and experience survey at the check-out visit and at the 12-months follow up when participants return for their annual DR screening using POC-AI. Surveys will be available in English and Spanish **(Appendix 6).**
2. **Diabetic retinopathy screening.** the screening will be conducted in a private office at each clinic study site (Chula Vista and King-Chavez) utilizing a Topcon TRC-NW400 Non-Mydriatic Retinal Camera that integrates an advanced, automated fundus camera capable of capturing true color images. It features a 360º rotating touch panel monitor, allowing the operator (staff) to capture images quickly and easily with a simple finger touch. The process also involves the EyeArt software, which is the first and only FDA-cleared AI technology capable of autonomous detection of both more than mild(mtm) and vision-threatening (vt) DR **(Appendix 3).** [19-20,24] The EyeArt AI software will evaluate captured images to detect signs of DR within 60 seconds. Participants will receive the results as follows: normal, mtmDR, vtDR, or ungradable. The study staff will explain that the results of the screening are not the final diagnosis, and the results will be reviewed by their provider during the medical visit. The medical provider will review the results with the participant and discuss any recommended treatment plan, such as a referral to an eye specialist. Ultimately, it will be the participant's choice whether to proceed with additional tests or treatments to evaluate what was observed. The study staff will closely monitor the progress and follow up with participants with abnormal results. The results of the DR screening are also documented in participant’s medical chart.

We are requesting a partial waiver of individual HIPAA authorization to review existing medical records in efforts to identify potential research subjects. The use of disclosure of PHI involves no more than minimal risk and granting the waiver will not adversely affect privacy rights and the welfare of the individuals whose records will be used. The random sample of patients from the participating clinics could not practicably be done without the waiver, as access to patient records will be necessary to provide the random sample. When potential subjects are approached to participate, they will be presented with the HIPAA research authorization **(Appendix 7).** PHI will not be re-used or disclosed for other purposes and whenever appropriate, the subjects will be provided with additional pertinent information after participation.

## 8.2 SAFETY AND OTHER ASSESSMENTS

The methods of documenting consent/permission/authorization will be obtained before any study procedures are performed including screening procedures. The information communicated to the participant or legally authorized representative during the consent process will not include exculpatory language through which the participant or legally authorized representative is made to waive or appear to waive any of the participant’s legal rights or release or appear to release the Researcher, Sponsor, the Organization or its agents from liability for negligence. The risks of participation in the study outweigh the value of the information to be gained. The research team anticipates the study results and learnings will reduce the risk of severe vision loss and improve clinical outcomes for individuals who choose to

participate. The selected POC- AI for DR screening is FDA-approved and uses a camera system to obtain retina images without the need for dilation. Thus, implementation of the screening poses no more than minimal risk to the subject.

In addition, the DRES-POCAI study team has developed standard operating procedures and evaluations to monitor safety and the proper understanding of conducting this study with the highest standards of ethical guidelines and regulatory requirements to safeguard participant well-being and ensure the validity of study findings. The study leadership (PIs, Manager) will be in close communication with the and staff (CTC and RAs) to ensure they have received the comprehensive training and materials necessary to conduct the research activities (recruitment, eligibility screening, consenting, administration of surveys, DR screenings, check out and follow ups) according to the protocols. The study's leadership will ensure the following procedures and evaluation are conducted during the implementation of the study:

*Safety monitoring:* Study protocols include continuous monitoring of participant safety throughout the study duration, and regular assessments of adverse events and any potential risks associated with the intervention. The staff will be well trained to identify, report and manage any adverse events or safety concerns **(SOPs Informed Consent, Adverse Events and Quality Control and Quality Assurance)**.

*Eligibility Screening Assessments*: The study does not involve the collection or assessment of any biological samples or specific clinical or radiological procedures to determine study eligibility. However, it is important the staff complete the eligibility screening for potential participants and validate participants' eligibility based on the inclusion criteria. This process also involves the validation of the eligibility based on the exclusion criteria which involves pre-determined health conditions that are considered contraindications for the use of the using a special camera and EyeArt® system for the autonomous AI-based DR screening. Example of the contraindications are: persistent visual impairment in one or both eyes, history of macular edema, moderate non-proliferative retinopathy, severe non-proliferative retinopathy, proliferative retinopathy, or retinal vascular (vein or artery) occlusion, history of ocular injections, laser treatment of the retina, or intraocular surgery (other than uncomplicated cataract surgery), or individuals who are contraindicated for fundus photography (for example, have hypersensitivity to light) **(SOP Eligibility Screening).**

*Enrollment Procedures*. Study coordinator will conduct regular assessments and create reports to ensure that enrolled participants meet all study criteria, have provided informed consent, randomization and allocation of participants to the appropriate intervention groups was correct, and to validate participant's information, such as demographics, medical history, and other relevant information **(*SOPs Informed Consent, Randomization and Quality Control and Quality Assurance*).**

*Intervention components and procedures*. Study manager and coordinator will conduct regular assessments of participant adherence to the study intervention protocol and monitoring of participant compliance with study procedures and requirements during the intervention which includes the health education sessions, administration of surveys and the DR screening. The staff will also monitor participant response to the intervention and any observed changes in health status, for example abnormal results of the DR Screening, also described in this document in sections **2.3.2, 2.3.3** and **8.1. (*SOPs Surveys, Diabetic Retinopathy Screening and Participant Education and Quality Control and Quality Assurance*).**

- *Surveys/Questionnaires:* Some of the questions we ask may make participants feel uncomfortable. Study staff will emphasize that they are not obligated to answer any questions that make them uncomfortable, and that they can skip any question or stop participation at any time. The staff will make note of the instances when this situation happened, will report to the study leadership and document in the eCRF.

- *Diabetic Retinopathy Screenings*: Participants assigned to the POC-AI group may experience discomfort during the DR screening, such as light flashes or eye strain. It's important to note that these potential discomforts are minimal and momentary. The staff will track and report on the participants that decline or withdraw from the study for this reason. The staff will document this situation in the participant's eCRF and report to study leadership. Additionally, participants will not undergo dilation for the POC-AI DR screening, only participant in usual care group. The DR screening is part of a participant's medical care. While abnormal results may cause concern or stress, participants will receive information and clarify that these results do not constitute a final diagnosis. The study staff will explain that the results of the screening are not the final diagnosis, and the results should be reviewed by their medical provider. The medical provider will review the results with the participant and discuss any recommended treatment plan, such as a referral to an eye specialist. Ultimately, it will be the participant's choice whether to proceed with additional tests or treatments to evaluate what was observed.

*Intervention Data Collection and Monitoring, and Quality Assurance.* This is part of the Quality Control and Quality Assurance (QC/QA) protocols of the study. It includes the collection of study data related to safety outcomes, intervention efficacy, and participant characteristics. The study DA and leadership will also conduct regular monitoring of study data to ensure accuracy, completeness, and integrity. They will meet every week to review the weekly reports and discuss the progress of the intervention. In addition, the study coordinator will conduct observations and QC/QA audits to monitor study staff adherence to the study protocol and SOPs. The implementation of the study's QC/QA measures will ensure the overall integrity and reliability of study data and procedures. The study team will conduct regular audits and reviews to identify and address any potential issues implementing the corrective actions and training needed **(SOP *Quality Control and Quality Assurance*).**

*Data Safety and Monitoring Committee (DSMC)*. The study will also have an internal Data Safety and Monitoring Committee. The committee's members will include faculty from academic institutions (i.e., San Diego State University and UC San Diego) that are principal investigators or co-investigators of federally funded research projects, SYHealth medical experts with experience in clinical research, SYHealth quality department members with expertise in risk management, SYHealth quality analytics staff, and the coordinator of the SYHealth's Research Review Committee with extensive experience in Ethical and Responsible Conduct of Research and Human Subjects Protection programs. The purpose of the DSMC is to 1) periodically review and evaluate the accumulated study data for participant safety, study conduct and progress and efficacy, 2) assess all adverse events reported during the course of the study and 3) make recommendations concerning the continuation, modification, or termination of the study. The DSMB will consider study-specific data as well as relevant background knowledge about the disease, intervention, or target population under study.

## 8.3 ADVERSE EVENTS AND SERIOUS ADVERSE EVENTS

*Leave blank. Text should be included under the relevant subheadings below.*

### 8.3.1 DEFINITION OF ADVERSE EVENTS (AE)

The FDA definition of an Adverse event is any untoward medical occurrence associated with the use of an intervention in humans, whether or not considered intervention-related (21 CFR 312.32 (a)). Additionally, the study team defines an adverse event as any unfavorable medical occurrence in a subject related to research participation, including signs, symptoms, or diseases.

The DRES-POCAI study has a protocol that describes the procedures for identifying, monitoring, and reporting all AE, including both serious and non-serious events, and UP involving risk to participants or others. There are different categories of AE and UP that may occur with individuals who present at SYHealth enrollment sites with the intention of participating in the research study **(SOP Adverse Events and Unanticipated Problems).**

- **Potential Risk (PR):** The possibility of harm or negative outcomes that participants might experience as a direct or indirect result of their involvement in the study. These risks can be physical, psychological, or socioeconomic in nature.
- **Adverse Event (AE):** Any unfavorable medical occurrence in a subject related to research participation, including signs, symptoms, or diseases.
- **Serious Adverse Event (SAE):** An adverse event with severe outcomes like death, life-threating situations, extended hospitalization, persistent disability, or those needed medical intervention to prevent such outcomes.
- **Unanticipated Problem (UP):** An adverse event unexpected in the research context, possible related to participation, and indicating higher risks than previously known.

### 8.3.2 DEFINITION OF SERIOUS ADVERSE EVENTS (SAE)

An adverse event (AE) or suspected adverse reaction is considered "serious" if, in the view of either the investigator or sponsor, it results in any of the following outcomes: death, a life-threatening adverse event, inpatient hospitalization or prolongation of existing hospitalization, a persistent or significant incapacity or substantial disruption of the ability to conduct normal life functions, or a congenital anomaly/birth defect. Important medical events that may not result in death, be life-threatening, or require hospitalization may be considered serious when, based upon appropriate medical judgment, they may jeopardize the participant and may require medical or surgical intervention to prevent one of the outcomes listed in this definition. Examples of such medical events include allergic bronchospasm requiring intensive treatment in an emergency room or at home, blood dyscrasias or convulsions that do not result in inpatient hospitalization, or the development of drug dependency or drug abuse.

### 8.3.3 CLASSIFICATION OF AN ADVERSE EVENT

#### 8.3.3.1 SEVERITY OF EVENT

The study will also align its protocols with the standard definition of the AEs by severity of the situation or event.

- **Mild** – Events require minimal or no treatment and do not interfere with the participant's daily activities.
- **Moderate** – Events result in a low level of inconvenience or concern with therapeutic measures. Moderate events may cause some interference with functioning.
- **Severe** – Events interrupt a participant's usual daily activity and may require systemic drug therapy or other treatment. Severe events are usually potentially life-threatening or incapacitating. Of note, the term "severe" does not necessarily equate to "serious".

#### 8.3.3.2 RELATIONSHIP TO STUDY INTERVENTION

All AEs must have their relationship to study intervention assessed by the team member who conducted the intervention activities with the participant based on temporal relationship and staff judgment. The

degree of certainty about causality will be graded using the categories below. In a clinical trial, the study product must always be suspect.

- **Related** – The AE is known to occur with the study intervention, there is a reasonable possibility that the study intervention caused the AE, or there is a temporal relationship between the study intervention and event. Reasonable possibility means that there is evidence to suggest a causal relationship between the study intervention and the AE.
- **Not Related** – There is not a reasonable possibility that the administration of the study intervention caused the event, there is no temporal relationship between the study intervention and event onset, or an alternate etiology has been established.

#### 8.3.3.3 EXPECTEDNESS

The Data Safety and Monitoring Committee (DSMC) of the study will also be responsible for determining whether an AE is expected or unexpected. An AE will be considered unexpected if the nature, severity, or frequency of the event is not consistent with the risk information previously described for the study intervention.

### 8.3.4. TIME PERIOD AND FREQUENCY FOR EVENT ASSESSMENT AND FOLLOW-UP

The occurrence of an AE or SAE may come to the attention of study staff during study visits and interviews of a study participant or upon review by a study monitor. All AEs that do not meet the criteria for SAEs will be captured on the appropriate case report form (CRF). Information to be collected includes event description, time of onset, staff or expert's assessment of severity, relationship to study intervention (assessed only by those with the training and authority to make a decision), and time of resolution/stabilization of the event. All AEs occurring while on study must be documented appropriately regardless of relationship. The study manager will record all reportable events with start dates occurring any time after informed consent is obtained until 7 (for non-serious AEs) or 30 days (for SAEs) after the last day of study participation. At each study visit, the PI will inquire about the occurrence of AE/SAEs since the last visit. All AEs will be followed to adequate resolution **(SOP Adverse Events and Unanticipated Problems).**

The Point of Care Artificial Intelligence DR screening procedure presents minimal risk to participants and the RAs will emphasize that it is specifically for diabetic retinopathy, not a substitute for a full eye examination. Examples of anticipated AE and AE response.

- If a participant shows signs of depression, stress, or anxiety, the staff will approach each situation with caution. This includes being attentive during consent, baseline survey, or screening processes that might emotionally affect the participant. In such cases, the staff will offer the participant a break, provide tissues or water, and inform them of the remaining tasks, asking if they would like to continue or need a break.
- If a participant shows signs of syncope or near syncope, the staff will assist the participant to either sit with their head between their knees or recline, if possible, use smelling salts, if necessary, provide a basin and towel if the participant is feeling nauseous, and ensure the participant remains seated until recovery. The study coordinator will be notified and decide whether the participant should continue with study activities or if medical intervention is needed.

### 8.3.5 ADVERSE EVENT REPORTING

Study staff are tasked with promptly documenting incidents (AE or SAE) in the appropriate study's form and in the SYHealth's Incident Report. This report should detail the incident without disclosing any Health

Insurance Portability and Accountability Act (HIPAA) identifiers of the study participants. It should also include the site staff's actions, outcomes, and follow-up plans. The PI or study manager must report any incidents or adverse events in this study to SYHealth's Risk Management and to the DSMC within 48 hours. All AEs including SAEs will be reviewed by the DSMC and reported to the UCSD Human Research Protections Program and the assigned Risk Manager at SYHealth within 7 days of the event occurring and summarized in the annual progress report. Immediate reporting is required for SAE, injuries, or incidents for which medical costs are incurred, or breaches of Personal Health Information (PHI) confidentiality.

Such adverse events include but are not limited to:

- Mandatory reporting case
- Need to break confidentiality to report criminal (e.g., child maltreatment) behavior of a provider, care coordinator, or family member
- Inadvertent harm related to participation in the study
- Loss of data related to the trial for any reason.

#### 8.3.6 SERIOUS ADVERSE EVENT REPORTING

Study staff will use the same protocol listed in 8.3.5 also SOP Adverse Events and Unanticipated Problems.

In the event of an emergency, the study team's immediate priority is to ensure that the participant received urgent medical care at the site or the nearest medical facility. Emergency contact information is available in clinics. In any crisis, staff should promptly contact the study coordinator or manager. They will decide whether to involve on-site medical staff of call 9-1-1. Immediate action is prioritized for participant safety before their departure from the premises. All emergencies, regardless of severity, must be reported to the SYHealth Risk management department, to the DSMC, UCSD-Institutional Review Board (IRB) and documented in CRF.

If a participant shows signs of a mental health emergency such as suicidal ideation, homicidal tendencies, or intoxication, study staff are trained to exercise judgment and sensitivity in these situations, consulting clinical decision-makers is necessary. Each SYHealth enrollment site has trained personnel to handle physical and medical emergencies. Emergency contact information is readily available in clinics. In any crisis, staff should promptly contact the study coordinator, manager or on-site medical provider. Immediate action is prioritized for participant safety before their departure from the premises. All emergencies, regardless of severity, must be reported to the SYHealth Risk management department to the DSMC and to the UCSD IRB. Documentation of the incident must be available in the participant's CRF.

#### 8.3.7 REPORTING EVENTS TO PARTICIPANTS

**Not applicable**

#### 8.3.8 EVENTS OF SPECIAL INTEREST

**Not applicable**

#### 8.3.9 REPORTING OF PREGNANCY

**Not applicable**

## 8.4 UNANTICIPATED PROBLEMS

*Leave blank. Text should be included under the relevant subheadings below.*

### 8.4.1 DEFINITION OF UNANTICIPATED PROBLEMS (UP)

Study staff will use the same protocol listed in 8.3.5 also SOP Adverse Events and Unanticipated Problems.

The Office for Human Research Protections (OHRP) considers unanticipated problems involving risks to participants or others to include, in general, any incident, experience, or outcome that meets all of the following criteria:

- Unexpected in terms of nature, severity, or frequency given (a) the research procedures that are described in the protocol-related documents, such as the IRB-approved research protocol and informed consent document; and (b) the characteristics of the participant population being studied,
- Related or possibly related to participation in the research ("possibly related" means there is a reasonable possibility that the incident, experience, or outcome may have been caused by the procedures involved in the research), and
- Suggests that the research places participants or others at a greater risk of harm (including physical, psychological, economic, or social harm) than was previously known or recognized.

An UP is an adverse event unexpected in the research context, possible related to participation, and indicating higher risks than previously known.

### 8.4.2 UNANTICIPATED PROBLEM REPORTING

The investigator will report UPs to the reviewing IRB. The UP report will include the following information:

- Protocol identifying information: protocol title and number, PI's name, and the IRB project number
- A detailed description of the event, incident, experience, or outcome
- An explanation of the basis for determining that the event, incident, experience, or outcome represents an UP
- A description of any changes to the protocol or other corrective actions that have been taken or are proposed in response to the UP.

To satisfy the requirement for prompt reporting, UPs will be reported using the following timeline:

- UPs that are SAEs will be reported to the IRB and to the DSMC within 7 days of the investigator becoming aware of the event.
- Any other UP will be reported to the IRB and to the DSMC within 7 days of the investigator becoming aware of the problem.
- All UPs should be reported to appropriate institutional officials (as required by an institution's written reporting procedures), the supporting agency head (or designee), and the Office for Human Research Protections (OHRP) within 7 days of the IRB's receipt of the report of the problem from the investigator.

### 8.4.3 REPORTING UNANTICIPATED PROBLEMS TO PARTICIPANTS

Not applicable

# 9 STATISTICAL CONSIDERATIONS

## 9.1 STATISTICAL HYPOTHESES

To evaluate the implementation and effectiveness of the multicomponent AI clinical intervention DRES-POCAI and its hypothesis all analyses will be performed at a patient level. Initially SYH will compare the intervention and control groups to determine if there was a significant (H2.1) increase in retinal screening uptake in the intervention group (primary outcome) and a corresponding (H2.2) increased diagnosis of DR (secondary outcome). The survey exploratory analyses will be performed to assess relationships between each item in the (H2.3) education questionnaire and the screening behavior (i.e., the proportion of patients with completed DR screen). These analyses will be used to identify items most strongly related to barriers and/or compliance, and thus, help refine the tool and guide future implementation strategies.

- Primary Efficacy Endpoint(s):

  Primary **effectiveness** outcome — **DR screening completion**

  Null hypothesis: $H_0$: There will be no difference in diabetic retinopathy screening rates between the intervention and standard of care study groups.

  Alternative hypothesis: $H_a$: More participants in the intervention group will complete diabetic retinopathy screening compared to participants in the standard of care group.

- Secondary Efficacy Endpoint(s):

  Secondary **effectiveness** outcome — **DR diagnosis**

  Null hypothesis: $H_0$: There will be no difference in diabetic retinopathy diagnosis between the intervention and standard of care study groups. Alternative hypothesis: $H_a$: More participants in the intervention group will be diagnosed with diabetic retinopathy compared to participants in the standard of care group.

- Exploratory analysis — identify **knowledge, attitudes, and self-efficacy** factors associated with DR screening uptake. Analyses will be used to identify items most strongly related to barriers and/or compliance and guide future implementation strategies.

## 9.2 SAMPLE SIZE DETERMINATION

Sample size determination based on the primary outcome: Based on the hypothesis that SYHealth will see a minimum absolute screening increase of 10%, from 59% in the standard of care group to 69% in the intervention group of retinal screening uptake, and assuming an $\alpha$ of 0.05 (significance), a $\beta$ of 0.8 (power), the target enrollment for analysis is 772 participants (361 per arm). However, if after the initial refinement phase of the project it is determined that either a smaller or larger percent of the target population is currently completing DR screening we will adjust the study enrollment accordingly, to ensure we will have sufficient statistical power to evaluate the anticipated 10% increase in screening uptake. Assuming the screening rate of the target population will range from 45% to 65% (current estimate at 59%) the number of participants needed would range from 658 to 784 (current estimate 722). Anticipating up to a 10% attrition rate from consent to study intervention (participants who agree to participate and consent via phone, and either do not keep their appointment or who at the clinic appointment decide to withdraw from the study), we estimate the target enrollment will range from 732 to 872 (current estimate is 848 patients, 424 per arm) to ensure an analysis size of 722 participants (361 per arm) for a two independent sample Pearson chi*2* test. Additionally, among the 2,514 patients who underwent retinal screening within the previous 12 months at the participating SYHealth clinics, 11% were diagnosed with DR; therefore, based on a screening uptake increase of 10%, a corresponding 16% increase in DR diagnosis (secondary outcome, H2.2) is anticipated in the intervention arm.

## 9.3 POPULATIONS FOR ANALYSES

All randomized participants will be included using an intent-to-treat analysis approach. Baseline differences at the patient level and within each clinic will be tested to determine if random assignment was successful. Any characteristics that differ between randomized groups will be included as covariates in all subsequent analytic models. If any significant imbalance is detected for any baseline characteristics, a sensitivity analysis will be performed, and the unbalanced characteristics will be incorporated into regression outcome models. We will evaluate the effectiveness of the tool across subgroups/categories by including a risk group classification variable in our models (to determine if there are differences in outcomes by group). If these risk groups are determined to confound the relationship between intervention and outcome, we will then stratify our analysis.

Additionally, if during the refinement and operationalization phase of Aim 1, specific high-risk groups are identified, and it is determined that there is a need to evaluate outcome by risk-group, for example, telemedicine versus in-person visit participants, we will add an additional block permutation and stratify randomization by telemedicine status in addition to clinic site.

## 9.4 STATISTICAL ANALYSES

*Leave blank. Text should be included under the relevant subheadings below.*

### 9.4.1 GENERAL APPROACH

Descriptive Statistics: Categorical data will be presented as frequencies and percentages. Based on normality assessments, continuous data will be presented as means (standard deviations) or medians (interquartile range). Patient characteristics will be presented using descriptive statistics and compared between intervention and standard of care groups using two-sample t tests for continuous (normally distributed) variables and Pearson's chi-squared test will be used for binary and categorical variables.

Inferential Tests: The impact of the intervention will be assessed by comparing the outcome of interest between intervention and usual care groups using logistic or poison regression (depending on distribution of outcome) and adjusting for identified covariates and assessing study intervention arm as the primary independent variable. A significant level (p-value) of 0.05 will be used with 95% confidence intervals reported. Two-tailed tests will be conducted unless there is a strong a priori basis for a one-tailed test.

Covariates: Covariates will be assessed individually between intervention and usual care groups and by outcome of interest. We will use Hosmer and Lemeshow's covariate inclusion approach (purposeful selection at each modeling step) when developing models to ensure inclusion of biologically and statistically relevant variables.

Assumptions: Data will be checked for normality of distribution prior to analysis. If assumptions are violated, appropriate transformations will be applied, or non-parametric tests will be used.

### 9.4.2 ANALYSIS OF THE PRIMARY EFFICACY ENDPOINT(S)

**Primary Endpoint: Increase in Retinal screening completion (H2.1)**

**Measurement**: Boolean variable (Yes/No) indicating completion of retinal screen within the defined study period (60 days from randomization).
**Scale:** Binary
**Analysis:** Chi-squared test or logistic regression will be used to compare screening completion rates between intervention and usual care groups. Regression covariates may include demographics and baseline clinical factors. Covariate selection will aim for a parsimonious model to achieve clarity and avoid overfitting. We will evaluate the intervention effectiveness across subgroups/categories by including a risk group classification variable in our models (to determine if there are differences in outcomes by group). If these risk groups are determined to confound the relationship between intervention and outcome, we will then stratify our analysis.
**Results Presentation**: Adjusted odds ratios with 95% confidence intervals will be reported.
**Assumption Checks**: When logistic regression is used, the data will be assessed for linearity, absence of multicollinearity, and influential outliers.
**Populations:** All enrolled participants will be included in the analysis.
**Missing Data:** Multiple deletion or imputation methods will be used to handle missing data depending on the pattern of missingness, e.g. if entire surveys are missing if missingness is driven by specific items or response groups in the survey, and/or if the data is missing completely at random. We have allowed for a 10% dropout/withdrawal rate between enrollment and intervention, and thus can use pairwise or listwise deletion of up to 10% of participants and still maintain adequate power for analysis.
**Outliers/Non-adherence**: Data outliers will be assessed to ensure they are not the result of data entry or measurement error. True data outliers will be handled either by trimming, adjusting the weight of the outliers, or by using robust regression techniques. Non-adherence will be addressed by analyzing data per-protocol in addition to intent to treat.

### 9.4.3 ANALYSIS OF THE SECONDARY ENDPOINT(S)

**Secondary Endpoint 1**: Early-stage DR diagnosis and treatment (H2.2)
**Measurement**: Diagnosis of DR.
**Scale**: Categorical (vtDR and mtmDR positive, vtDR negative and mtmDR positive, vtDR and mtmDR negative, ungradable).
**Analysis**: Chi-squared test or logistic regression will be used to compare screening completion rates between intervention and usual care groups. Regression covariates may include demographics and baseline clinical factors. Covariate selection will aim for a parsimonious model to achieve clarity and avoid overfitting. We will evaluate the intervention effectiveness across subgroups/categories by including a risk group classification variable in our models (to determine if there are differences in outcomes by group). If these risk groups are determined to confound the relationship between intervention and outcome, we will then stratify our analysis.
**Results Presentation**: Adjusted odds ratios with 95% confidence intervals will be reported.
**Assumption Checks**: When logistic regression is used, the data will be assessed for linearity, absence of multicollinearity, and influential outliers.
**Populations**: All enrolled participants will be included in the analysis.
**Missing Data**: Multiple deletion or imputation methods will be used to handle missing data depending on the pattern of missingness, e.g. if entire surveys are missing if missingness is driven by specific items or response groups in the survey, and/or if the data is missing completely at random. We have allowed for a 10% dropout/withdrawal rate between enrollment and intervention, and thus can use pairwise or listwise deletion of up to 10% of participants and still maintain adequate power for analysis.

**Outliers/Non-adherence**: Data outliers will be assessed to ensure they are not the result of data entry or measurement error. True data outliers will be handled either by trimming, adjusting the weight of the outliers, or by using robust regression techniques. Non-adherence will be addressed by analyzing data per-protocol in addition to intent to treat.

**Exploratory Endpoints**: Knowledge, Attitudes, Self-efficacy, and Patient Satisfaction (H2.3)
**Measurement**: Outcomes representing participant knowledge, attitudes, self-efficacy, and satisfaction with study participation.
**Scale**: Multiple outcomes will be explored and may include nominal, binary, or categorical data.
**Analysis**: We will explore the association between various demographic and clinical characteristics and knowledge, attitudes, self-efficacy, and patient satisfaction. Given the exploratory nature of this endpoint, analysis will be used to refine intervention implementation methods, identify barriers to participation, and increase participant satisfaction.

### 9.4.4 SAFETY ANALYSES

No safety endpoints will be analyzed, however documented all adverse events will be presented as a table in the final report and will include date of adverse event, description of event, and any resolution/ follow-up/or final outcome of the event.

### 9.4.5 BASELINE DESCRIPTIVE STATISTICS

At the time of enrollment, patient-level data will be collected from the participants' EHR, such as patient demographics, clinical data, and date of previous screening. Additional patient-level data will be collected in self-administered questionnaires. Baseline characteristics will be compared between intervention and usual care groups using two-sample t tests for continuous variables and Pearson's chi-squared test will be used for binary and categorical variables.
If any significant imbalance is detected for any baseline characteristics, a sensitivity analysis will be performed, and the unbalanced characteristics will be incorporated into regression outcome models.

### 9.4.6 PLANNED INTERIM ANALYSES

**Not applicable.**

### 9.4.7 SUB-GROUP ANALYSES

Sex and race/ethnicity will be included in both the primary (DR screening completion) and secondary (DR diagnosis) initial regression models.

### 9.4.8 TABULATION OF INDIVIDUAL PARTICIPANT DATA

All data will be captured at the individual participant level and will be listed by measure. Repeated measurements will be listed by measure and time from enrollment.

### 9.4.9 EXPLORATORY ANALYSES

For the survey exploratory analyses will be performed to assess relationships between each item in the **(H2.3)** education questionnaire and the screening behavior (i.e., the proportion of patients with completed DR screening). Multiple deletion or imputation methods will be used to handle missing data and will be dependent on the pattern of missingness, e.g. if entire surveys are missing if missingness is driven by specific items or response groups in the survey, and/or if the data is missing completely at random. We have allowed for a 10% dropout/withdrawal rate between enrollment and intervention, and thus can use pairwise or listwise deletion of up to 10% of participants and still maintain adequate power for analysis. These analyses will be used to identify items most strongly related to barriers and/or compliance, and thus, help refine the approach and guide future implementation strategies.

# 10 SUPPORTING DOCUMENTATION AND OPERATIONAL CONSIDERATIONS

*Leave blank. Text should be included under the relevant subheadings below.*

## 10.1 REGULATORY, ETHICAL, AND STUDY OVERSIGHT CONSIDERATIONS

*11 Leave blank. Text should be included under the relevant subheadings below.*

### 10.1.1 STUDY DISCONTINUATION AND CLOSURE

The DRES-POCAI study may be temporarily suspended or prematurely terminated if there is sufficient reason to do so. Circumstances that could warrant suspension or termination include unexpected safety concerns for participants, demonstration of overwhelming efficacy that necessitates providing the superior intervention to all, evidence that the study intervention is unlikely to demonstrate the intended effect, insufficient compliance with study protocols, data quality issues, or decisions by the sponsor, funding agency, or regulatory bodies.

This study may be temporarily suspended or prematurely terminated if there is sufficient reasonable cause. If the study is prematurely terminated or suspended, the Principal Investigator (PI) will promptly inform study participants, the Institutional Review Board (IRB), and sponsor and will provide the reason(s) for the termination or suspension. Study participants will be contacted, as applicable, and be informed of changes to the study visit schedule.

A suspended study may resume once any concerns about safety, protocol compliance, and data quality are addressed to the satisfaction of the sponsor, IRB, and relevant regulatory agencies.

### 10.1.2 CONFIDENTIALITY AND PRIVACY

Participant confidentiality and privacy is strictly held in trust by the DRES-POCAI study investigators, staff, and the sponsor(s). This confidentiality is extended to cover the clinical information relating to participants. Therefore, the study protocol, documentation, data, and all other information generated will be held in strict confidence. No information concerning the study, or the data will be released to any unauthorized third party without prior written approval of the sponsor.

All research activities will be conducted in a private office.

The study monitor (e.g., Multi-Specialty Quality Review Committee - *MSQRC*), other authorized representatives of the sponsor, representatives of the IRB, regulatory agencies may inspect all documents and records required to be maintained by the investigator, including but not limited to, medical records

(office or clinic) and DR screening records for the participants in this study. The clinical study site will permit access to such records.

The study participant's contact information will be securely stored at each clinical site for internal use during the study. At the end of the study, all records will continue to be kept in a secure location for as long a period as dictated by the reviewing IRB, organization's policies, or sponsor requirements.

Research data collected will be coded with a participant's unique identifier, or number. Personal identifying data will not appear on any research data. As is true for all SYHealth, UC San Diego personnel, and any person(s) working on this research project will have undergone extensive orientation and training on issues regarding the maintenance and protection of confidentiality (e.g., sending confidential material to a community printer, using names while conducting phone interviews, etc.)

Protecting participant confidentiality is a fundamental principle of SYHealth and this study. The study will employ strict measures to safeguard sensitive health information. These include secure storage of all study records with access limited to authorized research personnel. For analysis and reporting, participant data will be de-identified, and unique study codes will be assigned. The link between codes and participant names will be kept in a separate, secure location and will be accessible only to selected SYHealth personnel (PIs, Co-I, and Program Manager). Data transmission to research partners will utilize secure protocols, and data will be de-identified prior to sharing. Any relevant details about study participation (e.g., screening results) will be added to the participant's SYHealth medical records to ensure continuity of care. In accordance with SYHealth policies and standard privacy laws, participants retain the right to access their medical records, but some research-specific information may be inaccessible to them (e.g., survey responses).

Study findings may be published or shared at conferences, but individual participant information will always remain confidential. De-identified data collected during the study might be used for future research or shared with other scientists, but no personal identifiers will be included. Participants will sign a HIPAA Research Authorization form to permit the use of their protected health information in this study. This also facilitates continuity of care related to any study outcomes.

### 10.1.3 FUTURE USE OF STORED SPECIMENS AND DATA

De-identified data collected during the study might be used for future research or shared with other scientists, but no personal identifiers will be included.

### 10.1.4 KEY ROLES AND STUDY GOVERNANCE

*Provide the name and contact information of the Principal Investigator and the Medical Monitor.*

| **Principal Investigator** | **Principal Investigator** | **Medical Monitor** |
|---|---|---|
| Fatima A. Munoz, MD, MPH Associate VP, Health Support Services | Nicole Stadnick, PhD, MPH, Assistant Professor | Sharon Velasquez, MD |
| San Ysidro Health | University of California, San Diego | San Ysidro Health |
| 1601 Precision Park, San Ysidro, CA, 92173 | 9500 Gilman Drive, La Jolla, CA 92093 | 1601 Precision Park, San Ysidro, CA, 92173 |
| 619-395-8455 | 858-966-7703 | 619-662-4100 ext. 3306 |
| fatima.munoz@syhealth.org | nstadnic@health.ucsd.edu | sharon.velasquez@syhealth.org |
| | | |

### 10.1.5 SAFETY OVERSIGHT

Safety oversight will be under the direction of a Data and Safety Monitoring Committee (DSMC) composed of individuals with the appropriate expertise, including faculty from academic institution (i.e., San Diego State University) that are leaders of federally funded research projects, SYHealth medical expert with experience clinical research, member of the quality department with expertise in risk management, member of the quality analytics team, and the coordinator of the SYHealth Research Review Committee with extensive experience in Ethical and Responsible Conduct of Research and Human Subjects Protection program at SYHealth. Members of the DSMC are independent from the study conduct and free of conflict of interest, in addition, measures will be in place to minimize perceived conflict of interest. The DSMC will meet at least semiannually to assess safety and efficacy data on each arm of the study. The DMSC will operate under the rules of an approved protocol that will be written and reviewed at the organizational meeting of the DSMC. At this time, each data element that the DSMC needs to assess will be clearly defined. The purpose of the DSMC of the DRES-POCAI study will be to 1) periodically review and evaluate the accumulated study data for participant safety, study conduct and progress and efficacy, 2) assess all adverse events reported during the course of the study and 3) make recommendations concerning the continuation, modification, or termination of the study. The DSMC will consider study-specific data as well as relevant background knowledge about the disease, intervention, or target population under study.

### 10.1.6 CLINICAL MONITORING

The DRES-POCAI study clinic sites monitoring will be conducted to ensure that the rights and well-being of trial participants are protected, that the reported trial data are accurate, complete, and verifiable, and that the conduct of the trial is in compliance with the currently approved protocol/amendment(s), with International Conference on Harmonization Good Clinical Practice (ICH GCP), and with applicable regulatory requirement(s), e.g., SYHealth clinical guidelines.

The Clinical Monitoring Protocol (CMP) establishes the guidelines for conducting monitoring visits and related tasks for monitoring Diabetic Retinopathy Screening Point-of-Care Artificial Intelligence - DRES-POCAI Protocol number 810204. The CMP was developed by San Ysidro Health Quality and data analytics

team, in collaboration with the Multi-Specialty Quality Review Committee (MSQRC) and the Principal Investigator (PI). The MSQRC group is a multi-disciplinary team composed of clinical experts in primary care and behavioral health, risk management, quality, and operations. Moreover, two of the team members are the Director of behavioral health services and the director of the residency program with extensive experience in clinical research. The Monitoring Team (Quality Manager and Data Analytics Manager) is part of the MSQRC with the main purpose to ensure the study is conducting in compliance with the clinical guidelines and SYHealth's policies and procedures for clinic operations and patient care (as applicable). The Monitoring team will be responsible for performing the monitoring tasks in accordance with the protocol specific requirements.

*__Monitoring Plan__*

The review and monitoring of the data will be conducted by the monitoring team. The areas of focus for the study internal audits are site assessment review and staff training complete (competency check list and certificates), Human subjects' protection, Study's protocols (SOPs) compliance (QC/QA competency assessment), regulatory compliance (SYHealth guidelines and policies), clinical quality assurance, adverse event reporting and overall integrity of research data and DR screening. The review will be conducted as follows:

a) Review of Consent Forms: Random selection (up to 90%) reviewed. Results of findings may lead to 100% review of consent forms.
b) Review reports on missed events, missing data, and protocol deviations for a determined sample of subjects.
c) Compare source documentation to ensure data is accurate and complete. Any information access limitations, or special circumstances with respect to involvement of a data management system, will be specified in the approved, study-specific CMP. The CMP will include the percentages and processes of data review by the monitor, including a discussion of electronic databases used to collect data and the scope of involvement if there is a separate Data Coordinating Center.
d) Visit Schedule. The review if the Site will be conducted by the quality team under the direction of the quality director. The site review will be conducted as follows:
    - Review of credentials, training records, and delegation of responsibility logs.
    - The first monitoring visit will be conducted within 3 months after the first subject is enrolled and/or as outlined in the approved, study-specific CMP.
    - Interim monitoring visits will take place every 6 months while the study is active and have subjects returning for follow-up visits. Ad hoc or for-cause monitoring visit as needed or requested.

*__Monitoring Reporting Plan__*

The Monitoring team will present the results of audits and assessments to the designated study leadership representative (either the PI or manager). Preliminary findings will be discussed at the conclusion of the visit, and the leadership will initiate appropriate actions to address any outstanding issues identified. Monitoring visit findings and resulting action items will also be documented by the study leadership in the study reports (within QC/QA study records) for follow up and tracking purposes. The Monitoring team will also send the reports to MSQRC within 14 calendar days of the last day of the monitoring visit, and MSQRC will send final comments back to the monitoring team within 14 calendar days. Once the visit documents are finalized, documents will be made available to discuss with the study leadership member. These documents should be printed and stored in the study's physical area. Ideally, documents will be provided to the DRES-POCAI leadership in approximately 4 weeks of visit conclusion. A Monitoring team designee will work with designated site staff to resolve any outstanding action items as communicated in the Action Item Tracker.

### 10.1.7 DATA HANDLING AND RECORD KEEPING

*Leave blank. Text should be included under the relevant subheadings below.*

#### 10.1.7.1 DATA COLLECTION AND MANAGEMENT RESPONSIBILITIES

Data collection is the responsibility of the study staff at the site under the supervision of the study PI. The PI is responsible for ensuring the accuracy, completeness, legibility, and timeliness of the data reported. All source documents should be completed in a neat, legible manner to ensure accurate interpretation of data. Hardcopies of the study visit worksheets may be provided for use as source document worksheets for recording data for each participant enrolled in the study. The DRES-POCAI study will use electronic data collection platform and study data will be recorded in the electronic case report form (eCRF) derived from source documents.

Clinical data (including AEs data) and research surveys will be entered into the Research Electronic Data Capture (REDCap) program, a secure HIPAA compliant and a 21 CFR Part 11-compliant web-based system data capture system provided by the SYHealth for the DRES-POCAI study. The data system includes password protection and internal quality checks, such as automatic range checks, to identify data that appear inconsistent, incomplete, or inaccurate. Clinical data will be entered directly from the source documents or systems.

*Data Collection:* Research staff at the site, under the supervision of the Program Manager and PI, are responsible for collecting study data. The PI is ultimately responsible for the accuracy, completeness, legibility, and timeliness of all reported data.
*Source Documents***:** Consent, surveys and other participant data collected during the intervention will primarily be carried out electronically. Hard copies of collection instruments will be available for participants who prefer them or as a backup in case of technological failure. Source documents, whether electronic or hard copies, must be completed neatly and legibly to ensure accurate data interpretation. Hardcopies of study visit worksheets may be utilized as source documents for recording participant data. Data entered into the electronic Case Report Form (eCRF) must be consistent with the source documents, regardless of whether the original source was electronic or paper-based.
*Data Entry***:** Clinical data, surveys and screening results data will be entered into REDCap, 21 CFR Part 11-compliant system. The system includes password protection and quality checks (e.g., range checks) to flag inconsistent or inaccurate data.
*Data Sources*: Data will be collected from a variety of sources:

- Participant Surveys: Baseline and follow-up surveys will be administered via EPIC-Research through MyChart (self-administered) or in paper format, if necessary.
- Electronic Health Records (EHR): Patient-level data including demographics, clinical data, DR screening results, referrals, diagnoses, and other relevant health measures will be extracted from the EHR.
- EyeArt® Point-of-Care AI System: Data from the POC-AI system (screening results, images, time metrics) will be collected.

#### 10.1.7.2 STUDY RECORDS RETENTION

Study documents will be retained for a minimum of 3 years following the formal discontinuation of the study intervention. Documents may be retained for a longer period, however, if required by local regulations, internal institutional policies, or to fulfill scientific needs. Data collected and research results derived from this study may be used in future research projects and shared with other scientists in a de-identified format to advance scientific knowledge. This use of information is outlined in the informed consent.

### 10.1.8 PROTOCOL DEVIATIONS

A protocol deviation is any noncompliance with the clinical trial protocol, International Conference on Harmonization Good Clinical Practice (ICH GCP), or Manual of Procedures (MOP) requirements. The noncompliance may be either on the part of the participant, the investigator, or the study site staff. As a result of deviations, corrective actions are to be developed by the site and implemented promptly.

These practices are consistent with ICH GCP:

- 4.5 Compliance with Protocol, sections 4.5.1, 4.5.2, and 4.5.3
- 5.1 Quality Assurance and Quality Control, section 5.1.1
- 5.20 Noncompliance, sections 5.20.1, and 5.20.2.

It is the responsibility of the principal investigator (PI) to use continuous vigilance to identify and report deviations within 7 working days of identification of the protocol deviation, or within 7 working days of the scheduled protocol-required activity. All deviations must be addressed in study source documents, reported to Augmented Intelligence in Medicine and Healthcare Initiative (AIM-HI) – Kaiser Permanente Coordinating Center. Protocol deviations must be sent to the reviewing UC San Diego Institutional Review Board (IRB) per their policies. The PI is responsible for knowing and adhering to the reviewing IRB requirements.

The DRES-POCAI team is committed to minimizing protocol deviations through careful study design, staff training, and ongoing monitoring. However, in the event a deviation occurs, the following procedures will be followed:

*Identification and Reporting:*

- The study coordinator, manager and PI are responsible for identifying potential deviations promptly, whether related to participant actions, study procedures, or other factors.
- Deviations will be documented in the study QC/QA reporting system and discussed with the DRES-POCAI leadership (PI and manager) within one working day. The PI should report the deviation to the DSMC as well.
- Deviations that meet the criteria for reporting to the IRB will be submitted in a timely manner.

*Assessment and Classification:*

- The DRES-POCAI leadership will assess each deviation to determine its potential impact on participant safety, data integrity, and study outcomes.
- Deviations may be classified as:
    - Minor: Minor administrative errors or omissions that are unlikely to have a significant impact.
    - Major: Deviations that could potentially compromise participant safety or significantly affect data reliability.

*Corrective Actions:*

- Corrective actions (CA) will be developed in collaboration with the study team, addressing the root cause of the deviation to prevent recurrence.
- Preventive actions (PA) may also be implemented to avoid similar deviations in the future.
- CA and PA will be documented, and their implementation will be monitored.

*Analysis:*

- The Statistical Analysis Plan will outline how protocol deviations will be considered in the data analysis.

## 10.2 ADDITIONAL CONSIDERATIONS

**Not applicable**

## 10.3 ABBREVIATIONS

*The list below includes abbreviations utilized in this application.*

| | |
|---|---|
| AE | Adverse Event |
| ANCOVA | Analysis of Covariance |
| CFR | Code of Federal Regulations |
| CLIA | Clinical Laboratory Improvement Amendments |
| CMP | Clinical Monitoring Plan |
| COC | Certificate of Confidentiality |
| CONSORT | Consolidated Standards of Reporting Trials |
| CRF | Case Report Form |
| CTC | Clinical Trials Coordinator |
| DA | Data Analyst |
| DCC | Data Coordinating Center |
| DSMC | Data Safety Monitoring Committee |
| DRES-POCAI | Diabetes retinopathy Screening Point of Care Artificial Intelligence |
| eCRF | Electronic Case Report Forms |
| FDA | Food and Drug Administration |
| GCP | Good Clinical Practice |
| HIPAA | Health Insurance Portability and Accountability Act |
| IRB | Institutional Review Board |
| ICH | International Conference on Harmonization |
| ICMJE | International Committee of Medical Journal Editors |
| IDE | Investigational Device Exemption |
| IND | Investigational New Drug Application |
| IRB | Institutional Review Board |
| MOP | Manual of Procedures |
| MSQRC | Multi-Specialty Quality Review Committee |
| NCT | National Clinical Trial |
| NIH | National Institutes of Health |
| OHRP | Office for Human Research Protections |
| PI | Principal Investigator |
| PM | Program Manager |
| QA | Quality Assurance |
| QC | Quality Control |
| RA | Research Assistant |
| SAE | Serious Adverse Event |
| SAP | Statistical Analysis Plan |
| SMC | Safety Monitoring Committee |
| SOA | Schedule of Activities |
| SOP | Standard Operating Procedure |
| UP | Unanticipated Problem |
| US | United States |

## 10.4 PROTOCOL AMENDMENT HISTORY

*The table below is intended to capture changes of IRB-approved versions of the protocol, including a description of the change and rationale. A Summary of Changes table for the current amendment is located in the Protocol Title Page.*

| Version | Date | Description of Change | Brief Rationale |
|---|---|---|---|
| 1.0 | 05/07/24 | Document created | |
| 1.1 | 10/18/24 | Included $25 gift card | Added $25 compensation to recognize time and time invested in participation, and incentivize retention activities |
| 1.2 | 12/19/25 | Include active patients with upcoming visits and immediately following completed appointments. | In section 8.1EFFICACY ASSESSMENTS in page 20, a revision was made to expand recruitment by identifying active patients with a medical visit recommended as part of the standard of care not only patients with medical visit already scheduled at the time of the study visit. |
| | | | |
| | | | |
| | | | |
| | | | |
| | | | |
| | | | |
| | | | |
| | | | |
| | | | |
| | | | |
| | | | |
| | | | |
| | | | |
| | | | |
| | | | |
| | | | |
| | | | |

# 11 REFERENCES


1. Elsayed NA, Aleppo G, Aroda VR, et al. Retinopathy, Neuropathy, and Foot Care: Standards of Care in Diabetes—2023. Diabetes Care. 2023;46. doi:10.2337/dc23-S012
2. Flaxel CJ, Adelman RA, Bailey ST, et al. Diabetic Retinopathy Preferred Practice Pattern®. Ophthalmology. 2020;127(1). doi:10.1016/j.ophtha.2019.09.025
3. Fairless E, Nwanyanwu K. Barriers to and Facilitators of Diabetic Retinopathy Screening Utilization in a High-Risk Population. J Racial Ethn Heal Disparities. 2019;6(6). doi:10.1007/s40615-019-00627-3
4. Curran DM, Kim BY, Withers N, Shepard DS, Brady CJ. Telehealth Screening for Diabetic Retinopathy: Economic Modeling Reveals Cost Savings. Telemed J E Health. 2022 Sep;28(9):1300-1308. doi: 10.1089/tmj.2021.0352. Epub 2022 Jan 24. PMID: 35073213; PMCID: PMC9508450.
5. Integrating Eye Health and Vision Care for Underserved Populations into Primary Care Settings , American Optometric Association and the Association of Clinicians for the Underserved with support from The Centene Foundation for Quality Health Care , Dec. 2020, clinicians.org/wp-content/uploads/2020/11/Integrating-Eye-Health-and-Vision-Care-FINAL.pdf.
6. Chan AX, McDermott Iv JJ, Lee TC, Ye GY, Shahrvini B, Radha Saseendrakumar B, Baxter SL. Associations between healthcare utilization and access and diabetic retinopathy complications using All of Us nationwide survey data. PLoS One. 2022 Jun 15;17(6):e0269231. Doi: 10.1371/journal.pone.0269231. Erratum in: PloS One. 2023 Apr 27;18(4):e0285302. PMID: 35704625; PMCID: PMC9200294.
7. Meng YY, Diamant A, Jones J, Lin W, Chen X, Wu SH, et al. Racial and ethnic disparities in diabetes care and impact of vendor-based disease management programs. Diabetes Care. 2016;39(5):743–9
8. "Closing the Gap in Health Centers' Primary Eye Care." American Optometric Association , American Optometric Association Health Policy Institute, 19 Mar. 2019, www.aoa.org/news/inside-optometry/aoa-news/hpi-health-centers?sso=y.
9. Mukamal, Reena. "The Power of Ophthalmology in Community Health - Six Experts Discuss Innovative Programs That Are Improving Access and Care." American Academy of Ophthalmology, Aug. 2023, https://www.aao.org/eyenet/article/the-power-of-ophthalmology-in-community-health. Accessed 27 Sept. 2023.
10. Feldman H, ElSayed NA, McCoy RG, et al. Standards of Care in Diabetes—2023 Abridged for Primary Care Providers. Clin Diabetes. 2023;41(1). doi:10.2337/cd23-as01
11. Bhaskaranand M, Ramachandra C, Bhat S, et al. The Value of Automated Diabetic Retinopathy Screening with the EyeArt System: A Study of More Than 100,000 Consecutive Encounters from People with Diabetes. Diabetes Technology & Therapeutics. 2019;21(11). doi:10.1089/dia.2019.0164
12. Berkowitz ST, Finn AP, Parikh R, Kuriyan AE, Patel S. Ophthalmology Workforce Projections in the United States, 2020-2035. Ophthalmology. 2023;0(0). doi:10.1016/j.ophtha.2023.09.018
