# Supplementary material for: Diabetic Retinopathy Screening Among Federally Qualified Health Center Patients Using Point-of-Care AI: DRES-POCAI: A Trial Protocol
Source: JAMA Netw Open. 2025 Oct 21;8(10):e2538114. doi: 10.1001/jamanetworkopen.2025.38114 (PMC12541539; doi:10.1001/jamanetworkopen.2025.38114)
Supplement: Supplement 2. — Data Sharing Statement [file jamanetwopen-e2538114-s002.pdf]

Data Sharing Statement

Diaz. Diabetic Retinopathy Screening Among Federally Qualified Health Center Patients Using Point-of-Care AI. *JAMA Netw Open*. Published October 21, 2025.
doi:10.1001/jamanetworkopen.2025.38114

Data
Data available: Yes
Data types: Deidentified participant data
How to access data: The deidentified dataset will be available to external investigators upon approval of a scientific request. These requests must adhere to established San Ysidero Health guidelines and will be presented to the San Ysidro Health Research Review Committee, which will assess the proposed research for scientific merit and ethical considerations.
When available: With publication

Supporting Documents
Document types: Informed consent form, Other (please specify)
Additional Information: Protocol
How to access documents: The deidentified dataset will be available to external investigators upon approval of a scientific request. These requests must adhere to established San Ysidero Health guidelines and will be presented to the San Ysidro Health Research Review Committee, which will assess the proposed research for scientific merit and ethical considerations.
When available: With publication

Additional Information
Who can access the data: Researchers whose proposed use of the data has been approved
Types of analyses: San Ysidro Health Research Review Committee, which will assess the proposed research for scientific merit and ethical considerations.
Mechanisms of data availability: San Ysidro Health Research Review Committee, which will assess the proposed research for scientific merit and ethical considerations.
